# Liquid lens based holographic camera for real 3D scene hologram acquisition using end-to-end physical model-driven network

**DOI:** 10.1038/s41377-024-01410-8

**Published:** 2024-02-29

**Authors:** Di Wang, Zhao-Song Li, Yi Zheng, You-Ran Zhao, Chao Liu, Jin-Bo Xu, Yi-Wei Zheng, Qian Huang, Chen-Liang Chang, Da-Wei Zhang, Song-Lin Zhuang, Qiong-Hua Wang

**Affiliations:** 1 School of Instrumentation and Optoelectronic Engineering, Beihang University, Beijing, 100191 China; 2 School of Optical-Electrical and Computer Engineering, University of Shanghai for Science and Technology, Shanghai, 200093 China

**Keywords:** Displays, Imaging and sensing

## Abstract

With the development of artificial intelligence, neural network provides unique opportunities for holography, such as high fidelity and dynamic calculation. How to obtain real 3D scene and generate high fidelity hologram in real time is an urgent problem. Here, we propose a liquid lens based holographic camera for real 3D scene hologram acquisition using an end-to-end physical model-driven network (EEPMD-Net). As the core component of the liquid camera, the first 10 mm large aperture electrowetting-based liquid lens is proposed by using specially fabricated solution. The design of the liquid camera ensures that the multi-layers of the real 3D scene can be obtained quickly and with great imaging performance. The EEPMD-Net takes the information of real 3D scene as the input, and uses two new structures of encoder and decoder networks to realize low-noise phase generation. By comparing the intensity information between the reconstructed image after depth fusion and the target scene, the composite loss function is constructed for phase optimization, and the high-fidelity training of hologram with true depth of the 3D scene is realized for the first time. The holographic camera achieves the high-fidelity and fast generation of the hologram of the real 3D scene, and the reconstructed experiment proves that the holographic image has the advantage of low noise. The proposed holographic camera is unique and can be used in 3D display, measurement, encryption and other fields.

## Abstract (web summary)

A holographic camera for acquiring high-fidelity holograms of real 3D scenes based on a liquid camera and an end-to-end physical model-driven network is proposed.

## Graphical abstract

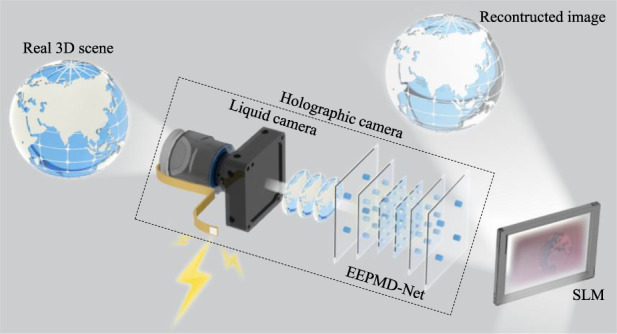

## Introduction

Holography can restore the complete light field information of the recorded object, which has great application value in the fields of data storage, biological microscopic imaging, optical micromanipulation and optical sensors^1–7^. An important frontier area of holography is realistic 3D scene reconstruction^8–10^. However, due to the huge computation of 3D objects in data representation and the influence of laser coherence, 3D holography has the following bottlenecks^11–15^: 1) It is difficult to quickly capture and reconstruct the real 3D scene with true depth. 2) The reconstructed image has serious speckle noise. These bottlenecks seriously hinder the development and application of 3D holography, and need to be broken through urgently.

Generally, before calculating the hologram of a real 3D scene, it is necessary to collect the data of the real 3D scene with a camera, then calculate the light field distribution of the real 3D scene, and finally generate the hologram by complex amplitude coding^16,17^. However, such a process is cumbersome and costly because the front-end data acquisition often takes a lot of time. Although in digital holography it is possible to directly capture the interference fringes of a real 3D scene with a camera, such a hologram acquisition approach is usually utilized in the fields of microscopic imaging and interferometry^18–21^. Digital holography needs to use laser to actively illuminate the real 3D scene, which prevents the miniaturization and integration of the system. As an adaptive optical element, electrowetting-based liquid lens has the advantages of fast response speed and adjustable focal length, and is widely used in scene acquisition^22–26^. Some researchers put forward the holographic near-eye display technology based on liquid lens, but the quality of reconstructed images is affected due to the limitation of liquid lens aperture^27^. The maximum aperture of the existing commercial electrowetting-based liquid lens is only 5.8 mm, which leads to the limited luminous flux and field of view in the process of light field transmission^28,29^.

For the hologram generation, 3D holography has made remarkable progress in high fidelity and rapid reconstruction of scenes in recent years with the development of neural network technology^30–33^. For example, the TensorHolo network is proposed to realize the rapid generation of holograms on smart phones^34^. In order to generate hologram of 3D scene, the end-to-end training network based on neural network preprocessing is further proposed^35,36^. In addition, some researchers propose a model-driven deep learning network, and the effect of high-fidelity holographic reconstruction can be realized^37–39^. Although some progress has achieved holographic reconstruction of real scenes, most of them only obtain 2D scene images, and then reconstruct 3D scene through relative depth estimation^40–43^. These technologies are hard to break through in the reconstruction of true depth, and the calculation speed and reconstruction quality need to be improved. Therefore, it is necessary to develop a miniature holographic camera that does not rely on coherent illumination light, so as to realize the fast acquisition of real-world holograms from the 3D scene capture.

**Fig. 1:**
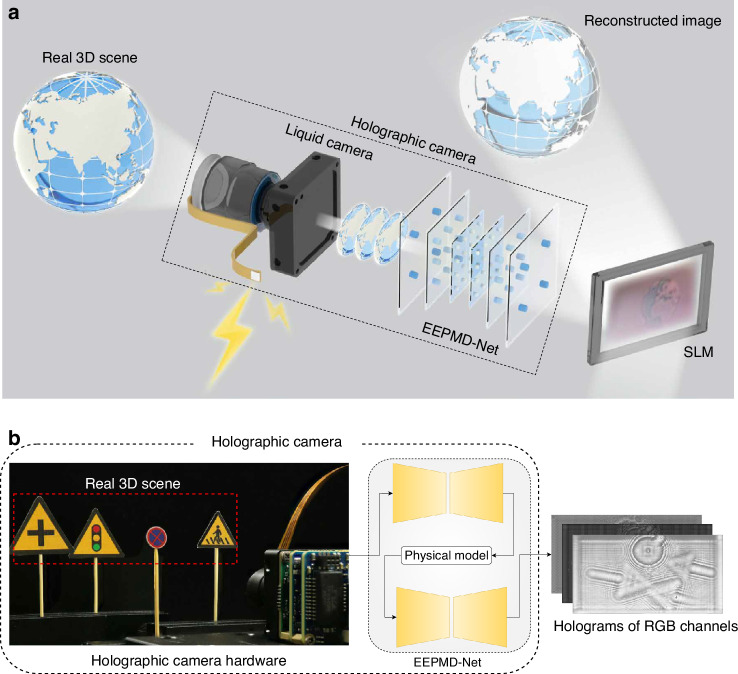
Schematic diagram of the holographic camera. **a** Concept map from real scene to holographic 3D reconstruction**. b** Structure of the proposed holographic camera

To solve the above problems, a holographic camera for obtaining high-fidelity hologram of real 3D scene is proposed in this paper, as shown in Fig. 1a. Different from the conventional cameras, the hardware module of the holographic camera employs a liquid camera based on a large-aperture liquid lens to quickly acquire the real 3D scene. To our knowledge, this is the first time to realize such a large size liquid lens through the matching of two phases without aqueous solution, so that the true depth of the high-quality 3D scene can be obtained within 100 ms. As the software part of the holographic camera, an end-to-end physical model-driven network (EEPMD-Net) is designed to generate the hologram of real 3D scene captured by the liquid camera. The full-focused image and depth map of the 3D scene are input to the EEPMD-Net. In the proposed EEPMD-Net, the encoder and decoder networks based on receptive field block and compound convolution algorithm are proposed to realize low noise phase calculation. By comparing the intensity information between the depth-fused reconstructed image and the target scene, the composite loss function is constructed to optimize the hologram phase. The red, green, and blue (RGB) holograms of the real 3D scene can be calculated in parallel within 53 ms based on the EEPMD-Net. When the holograms are loaded on a spatial light modulator (SLM) for reconstruction, the peak signal-to-noise ratio (PSNR) of the color reconstructed image can reach ~28 dB. The proposed holographic camera solves the two bottleneck problems of difficult scene acquisition and low reconstruction quality in existing holography, provides a new idea for high-fidelity 3D reconstruction of real scenes, and is expected to find new applications in many fields such as 3D display, measurement encryption and so on.

## Structure and principle

### Structure of the proposed holographic camera

The proposed holographic camera consists of a hardware based on the liquid camera and a software module based on the EEPMD-Net, as shown in Fig. 1b. The liquid camera is used to quickly capture the depth information of multiple focal planes of a real 3D scene. If the captured 3D scene information is directly used for diffraction calculation, it will be impossible to obtain high-fidelity holograms. For this reason, the EEPMD-Net is proposed to be the software module of the holographic camera. Based on the designed image fusion and depth calculation methods, the EEPMD-Net is able to process the information of a real 3D scene with multi-focal planes into a full-focused image and a depth map. Driven by the physical model and utilizing the trained encoding and decoding networks, the EEPMD-Net can quickly calculate the holograms of RGB channels of the real 3D scene. The corresponding relationship between the driving voltage of the liquid lens and the true depth can be established in the shooting process of the liquid camera. Therefore, by reasonably setting the parameters of the EEPMD-Net, high-fidelity holograms of the real 3D scene can be obtained.

### Design of the liquid camera based on liquid lens

The liquid camera is the core device of the hardware of the holographic camera (supplementary information S1). The liquid lens is used for focal length adjustment, and the solid lens group supports the main optical power. The total optical power of the liquid camera *Φ* can be expressed as follows:

1 $${\Phi }={{\Phi }}_{s}+{{\Phi }}_{1}-{d}_{s1}{{\Phi }}_{s}{{\Phi }}_{1}$$

where *Φ*_s_ and *Φ*_l_ are the optical powers of the solid lens group and the liquid lens respectively, and *d*_sl_ is the distance between the optical principal planes of the liquid lens and the solid lens group. When the liquid camera is used to capture targets with different depths in the real scene, only the applied voltage needs to be adjusted, and no mechanical movement of any components is needed. This mode ensures the fast response and high-accuracy adjustment of the liquid camera.

In order to realize a liquid lens with large aperture and high stability, the following requirements should be met when we prepare two types of electrowetting liquid filling materials. The propane-1,3-diol is used as polar solvent to replace common water. In the case of dielectric failure, this solvent will not have electrolytic reaction in the conductive liquid of the liquid lens, thus preventing the generation of electric sparks and bubbles. In addition, tetrabutylammonium chloride (TBAC) with proper concentration is added as the solute of propane-1,3-diol, thus enhancing the conductivity of the conductive liquid and adjusting the refractive index and density. While ensuring density matching, 1-bromo-4-ethylbenzene mixed with ISOPAR^TM^ V fluid is used as the insulating liquid. This insulating liquid has very low surface tension and can match the surface energy of hydrophobic materials. Therefore, a large initial contact angle of the biphasic liquids is ensured, and the liquid lens is allowed to drive the liquid-liquid interface to move at an aperture of 10 mm while maintaining a low driving voltage. Moreover, the viscosity of the biphasic liquids needs to be moderate. Too low viscosity will lead to excessive oscillation of the liquid-liquid interface, while too high viscosity will lead to excessive damping, both of which will increase response time. By controlling various parameters of the biphasic liquids, the liquid lens with 10 mm large aperture has the fast response, low driving voltage and sufficient range of optical power variation. This lays a foundation for the liquid camera to achieve an ideal field of view, fast response, large focus range and high stability.

According to the electrowetting effect, when the voltage applied to the upper electrode and lower electrode varies, the contact angle between the liquid-liquid interface and the lens cavity changes, resulting in the change of the curvature of the liquid-liquid interface, thereby altering the optical power of the lens. The relationship between the electrowetting contact angle *θ* and the applied voltage *V* can be described based on the Young-Lippmann equation. Then, the optical power *Φ*_l_ of the liquid lens can be calculated:

2 $${\Phi }_{1}=\frac{\left({2\gamma }_{{\rm{ci}}}\cos {\theta }_{0}+{{CV}}^{2}\right)\left({n}_{2}-{n}_{1}\right)}{{2\gamma }_{{\rm{ci}}}D}$$

where *γ*_ci_ is the interfacial tension between the conductive liquid and the insulating liquid, *θ*_0_ is the initial contact angle when no voltage is applied, *C* is the total capacitance per unit area of the dielectric layer and the hydrophobic layer, *D* is the aperture of the liquid lens, *n*_1_ and *n*_2_ are the refractive indexes of the conductive liquid and the insulating liquid, respectively.

### Principle of the EEPMD-Net

**Fig. 2:**
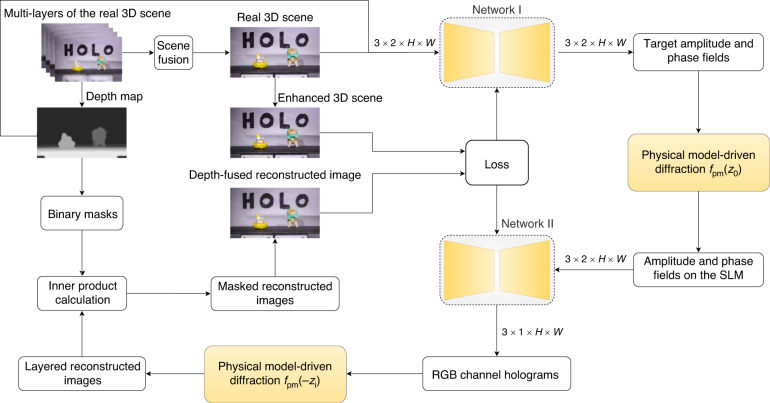
Structure and mechanism of the EEPMD-Net

The structure and mechanism of the proposed EEPMD-Net are shown in Fig. 2. The input of the EEPMD-Net is the full-focused image and depth map of the real 3D scene, and the output is the holograms of RGB channels of the real 3D scene. The EEPMD-Net includes five steps from the acquisition of real 3D scene to hologram generation.

Firstly, by using the proposed scene fusion and depth calculation method, the multi-layers of the real 3D scene captured by the liquid camera are processed into the full-focused image and the depth map of the real 3D scene. Each color of the full-focused images is connected with the depth map of the real 3D scene in the channel dimension, and the size of the concatenated image tensor is 3×2×*H*×*W* (where *H* is the height and *W* is the width). Secondly, the concatenated image tensor is input into network I. Network I is used to encode the concatenated image tensor and output the target amplitude and phase field of the real 3D scene with the tensor size of 3×2×*H*×*W*. Thirdly, a physical model *f*_pm_ is chosen and the target amplitude and phase fields of the real 3D scene are propagated forward by a distance of *z*_0_, resulting in the amplitude and phase fields on the SLM. Then, the complex amplitude on the SLM (with a tensor size of 3×2×*H*×*W*) is input into network II. Network II is used to encode the hologram of the real 3D scene and output the holograms of RGB channels with a tensor size of 3×1×*H*×*W*. Finally, the physical model *f*_pm_ is used to back-propagate the hologram by the distance of *z*_i_ (*i* = 1, 2,…) to get the reconstructed image of the *i*th layer of the real 3D scene. After each color channel of the full-focused image of the real 3D scene is trained, the RGB channel holograms can be generated quickly.

It should be noted that three color channels of the full-focused images of the real 3D scene are trained simultaneously. When calculating the loss of the EEPMD-Net, the comparison between the intensity information of the depth-fused reconstructed image and the enhanced 3D scene is obtained by using an unsharp mask filter (supplementary information S2.1). There are three steps to obtain the depth-fused reconstructed image. Firstly, the depth map is segmented according to the gray value and layers of the depth map, and binary masks with different depths are obtained. Then, the inner product of the binary mask and the layered reconstructed image at the same depth is calculated to obtain the masked reconstructed image. Finally, the masked reconstruction images of each layer are summed pixel by pixel to obtain the depth-fused reconstructed image. By using the constraints between the intensity information of the depth-fused reconstructed image and the enhanced 3D scene, the speed of the EEPMD-Net to calculate high-fidelity hologram is optimized. The EEPMD-Net contains network I and network II which are composed of the down-sampling block, receptive field block, up-sampling block, parameter rectified linear unit functional layer, and tangent hyperbolic functional layer (supplementary information S2.2–S2.4).

In the EEPMD-Net, the band-limited angular spectrum method is used to simulate the propagation of light waves (supplementary information S2.5). The band-limited angular spectrum method solves the numerical errors problem of the traditional angular spectrum method for far-field propagation. This is achieved by limiting the bandwidth of the propagating optical field and eliminating excess high-frequency information. Moreover, in order to make the generated image visually closer to the target image while retaining more details and texture information, a composite loss function consisting of perceptual loss, multiscale structural similarity loss, total variance loss and mean-square error loss is used in the EEPMD-Net (supplementary information S2.6).

## Results

### Fabrication and performance test of the liquid camera

**Fig. 3:**
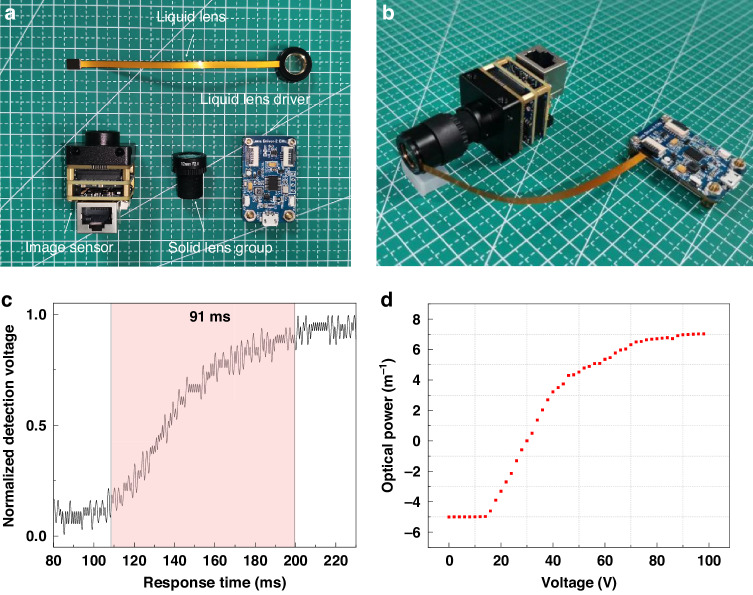
Fabrication of the liquid camera and performance test of the liquid lens. **a** Component of the liquid camera. **b** Photo of the liquid camera. **c** Response time of the liquid lens. **d** Optical power of the liquid lens under different applied voltages

The liquid camera is integrated firstly with a self-fabricated liquid lens, a solid lens group, a self-developed liquid lens driver and an image sensor, as shown in Fig. 3a, b. The focal ratio of the solid lens group (M12-HF12, Shenzhen Jinghang Technology Co., Ltd) is 2.8, the focal length is 12 mm, and the mechanism length is approximately 19 mm. A photosensitive chip with the type of Sony IMX178 is used in the image sensor. The size of the sensor area is 1/1.8”, and the pixel size is 2.4 μm. The liquid lens driver is self-developed based on STM32G070 and it can provide sufficient driving voltage for the liquid lens with a voltage adjustment step of ~0.2 V. As for the liquid lens, the material of the electrodes is aluminum, and the inner surface of the upper electrode is coated with a dielectric layer (Parylene C, ~3.15 dielectric constant, ~3 μm thickness) and a hydrophobic layer (Teflon^TM^ AF 2400, ~100 nm thickness). Between the upper and lower electrodes, a plastic spacer is employed to separate them, ensuring effective insulation between the lower and upper electrodes. Equal volumes of conductive liquid and insulating liquid are sequentially injected, and meticulous sealing is achieved with a two-component epoxy resin.

The specific parameters of the proposed biphasic liquids are shown in Table 1. The flexible electrodes are connected to the upper and lower electrodes using conductive silver paste and then assembled into the plastic shell. To achieve an ideal field of view for the liquid camera, the liquid lens is designed with a large aperture and a relatively small thickness. Through simulation and optimization, we manage to reduce the thickness of the liquid lens to just 6 mm while maintaining a 10 mm aperture, resulting in a diameter-to-thickness ratio of 1.67. The liquid lens and solid lens group are riveted through a custom-made housing, and the liquid lens is closely attached to the solid lens group. In addition, in the visible light band, the transmission of the proposed biphasic non-aqueous electrowetting liquids is always greater than 95%, and the total transmission of the liquid lens is greater than 90%, including window glass (supplementary information S3.1).

**Table 1:** Parameters of the proposed biphasic liquids

Parameters	Conductive liquid	Insulating liquid
Density (g/cm^3^)	1.048	1.048
Refractive index	1.4386	1.4902
Dynamic viscosity (mPa·s)	44.56	2.27
Conductivity (μS/m)	73	-
Interfacial tension (mN/m)	17.7	

After testing, the response time of the fabricated liquid lens is 91 ms and the range of optical power is −5 m^−^^1^ ~ 7.03 m^−^^1^. The relationship between response time and voltage and the relationship between optical power and voltage of the liquid lens are shown in Fig. 3c, d, respectively (supplementary information S3.2). The resolution and magnification of the liquid camera at different depths with different voltages are recorded during the shooting of the real 3D scene, which is used as the basis for depth calculation (supplementary information S3.3).

### Image capture and depth calculation

**Fig. 4:**
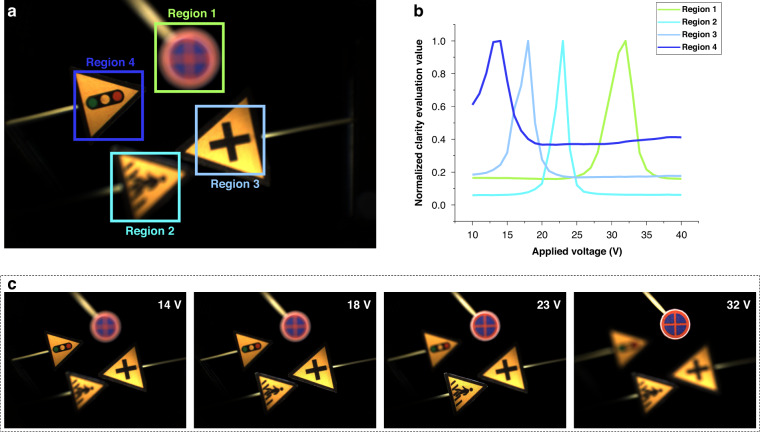
Image acquisition and clarity evaluation. **a** Regions of interest for the target signs. **b** Normalized CEVs for each region. **c** Images captured under different voltages

The liquid camera is used to capture a real 3D scene containing four signs. Benefiting from the rapid response of the liquid lens, the images with different focusing depths are quickly captured by simply changing the driving voltage. Four regions of interest are marked, each with a square area of 550×550 pixels, as shown in Fig. 4a. The images are captured under the driving voltages from 10 V to 40 V, and the magnifications of the images are corrected. The purpose of magnification correction is to prevent the targets from escaping from the marked regions due to the lens focus breathing effect, ensuring consistency in clarity comparison under different driving voltages. Then, the clarity evaluation value (CEV) of the marked regions is calculated by implementing convolution of images and Laplacian operator. Figure 4b shows the normalized CEVs of the four marked regions under different driving voltages. By fitting the CEV curves with the resolution data (supplementary information S3.4), the depths of the main target signs within the marked regions can be obtained. The depth values of the four regions are calculated as 100 mm, 150 mm, 200 mm and 250 mm, respectively, which are consistent with the actual settings.

Figure 4c shows the images captured when the maximum CEVs of the marked regions are reached respectively, and it can be seen that the focused images of the target signs are clearly captured. After obtaining focused images and the depth of the target sign, it is necessary to extract the complete shape of the target sign. The mask generation method is developed to realize it (supplementary information S4.1). After extracting the complete shape of each target sign, the fused full-focused scene and depth map are obtained based on the setting layers (supplementary information S4.2). Then, the fused scene and depth map are input to the EEPMD-Net for hologram calculation.

### Holographic reconstruction

**Fig. 5:**
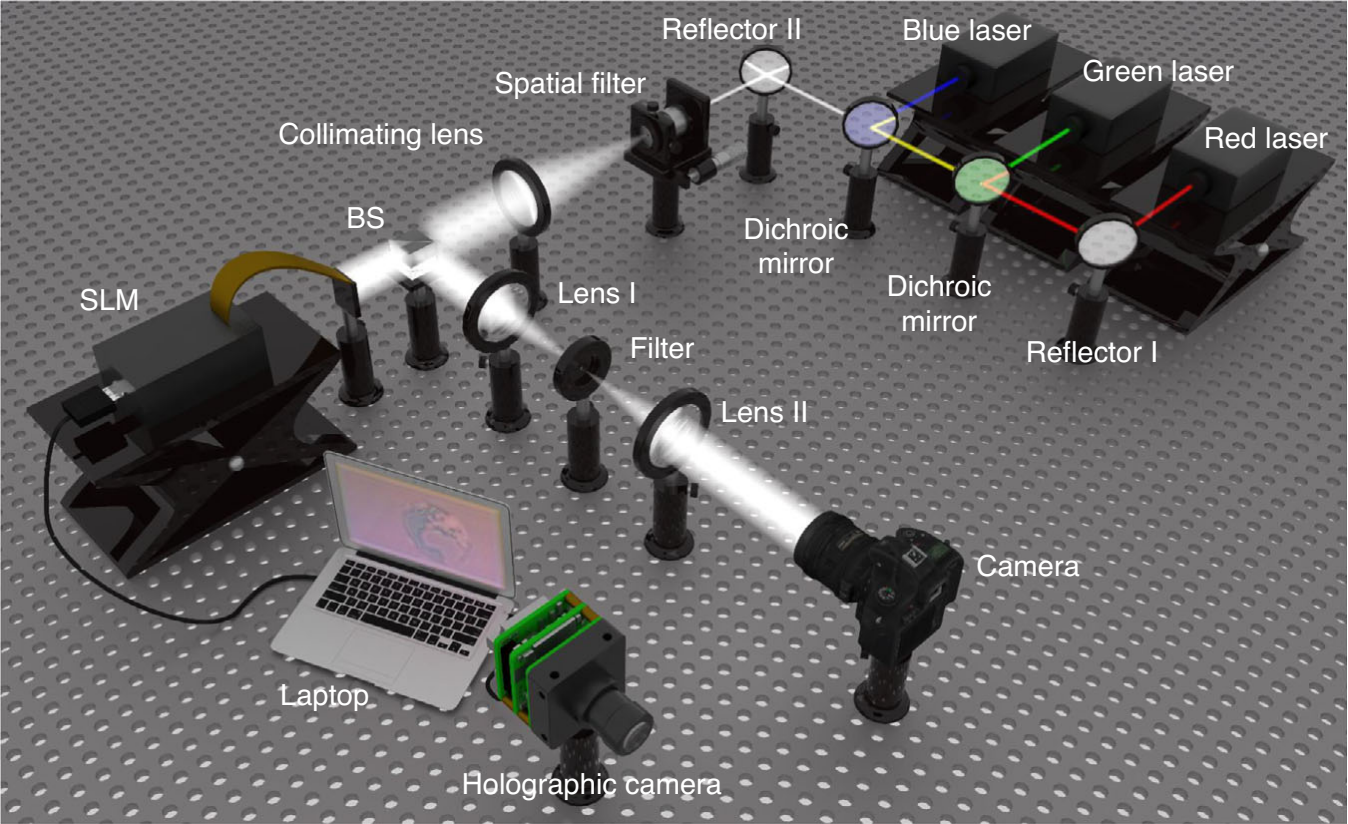
Structure of the reconstruction system

In order to verify the advantages of the proposed holographic camera, we transmit the hologram obtained by the holographic camera to the SLM for optical reconstruction, as shown in Fig. 5. The reconstruction system consists of a red laser, a green laser, a blue laser, two reflectors (including reflector I and reflector II), two dichroic mirrors, a spatial filter, a collimating lens, a beam splitter (BS), an SLM, a 4 *f* system (including a filter, lens I and lens II), a holographic camera, and a laptop. The wavelengths of the RGB lasers are 671 nm, 532.8 nm, and 471 nm, respectively. The resolution of the SLM manufactured by Xi’an CAS Microstar Science and Technology Co., Ltd. is 1920 × 1080 and its pixel pitch is 6.4 μm. The camera used to capture the reconstructed images is a Canon EOS 6D Mark II. The spatial filter and collimating lens are used to generate parallel light. Then the parallel light passes through the BS and illuminates the SLM. The 4 *f* system is used to eliminate the undesirable light. The three color holograms are loaded on the SLM in time sequence. When three color lasers illuminate holograms of corresponding colors respectively, color reconstructed image of the real 3D scene can be captured.

**Fig. 6:**
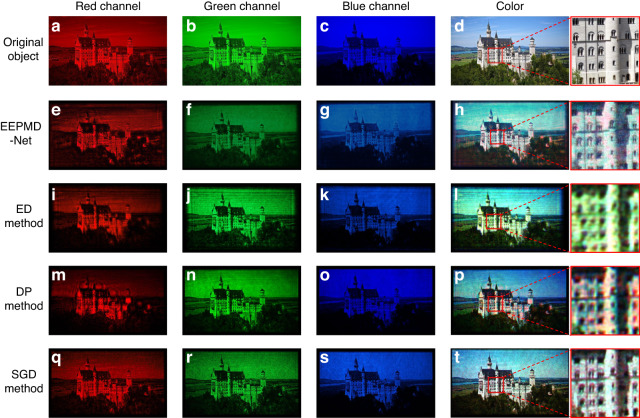
Comparison of the reconstructed images by using different methods. **a–d** Original object. **e–h** Reconstructed images using the EEPMD-Net. **i–l** Reconstructed images using the ED method. **m–p** Reconstructed images using the DP method. **q–t** Reconstructed images using the SGD method

In order to validate that the proposed EEPMD-Net can improve the quality of the holographic image, the error diffusion (ED) method, the double-phase (DP) method, and the stochastic gradient descent (SGD) method are used for comparison. The recorded object is a 2D object of a “castle” with a recording distance of 300 mm. The resolutions of the recorded object and the hologram are 990 × 1760 and 1072 × 1920, respectively. The color reconstructed images are shown in Fig. 6.

The “castle” obtained by the ED method shows the obvious ringing phenomenon, and the edges and local texture information of the “castle” are blurred and distorted. The “castle” obtained by the DP method has improved quality, and some of the edge information of the “castle” is preserved, but the ringing phenomenon still exists, and the local texture information is still unclear. The SGD method takes the longest calculation time, and the interfering stripes and speckle noise affect the clarity of the “castle”. The EEPMD-Net shows advantages in terms of calculation time and reconstruction quality, and the edges, details, and texture information of the “castle” are better preserved with few ringing phenomena and interference fringes. In addition, according to the comparison of the local enlarged images in Fig. 6, the color reconstructed image obtained by the EEPMD-Net is the closest to the original image, which indicates that it has significant advantages in improving the quality of reconstructed images (supplementary information S5.1).

**Fig. 7:**
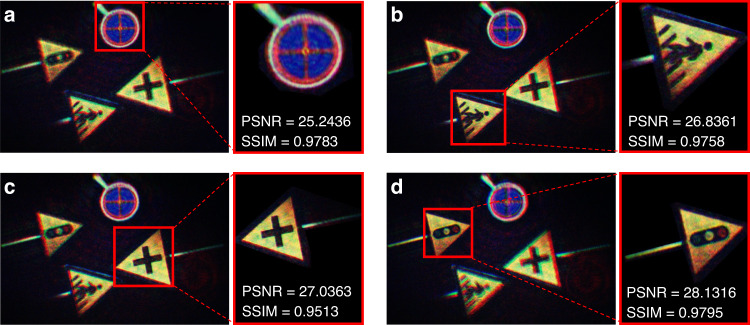
Experimental results of the color reconstructed images of the real 3D scene. **a–d** Reconstructed images when the “no parking”, “sidewalk”, “intersection” and “traffic light” signs are focused, respectively

Then the RGB holograms of the real 3D scene are calculated using EEPMD-Net. The resolutions of both the real 3D scene and the depth map are 1849 × 2773. The calculation times of the RGB holograms of the real 3D scene are 53 ms, 46 ms, and 27 ms, respectively. The color reconstructed images of the real 3D scene are shown in Fig. 7. The PSNR and structural similarity (SSIM, supplementary information S5.2) are used to evaluate the quality of the color reconstructed images. Four layers of signs (“no parking”, “sidewalk”, “intersection” and “traffic light”) are used as the real 3D scene. The actual spacing between the signs is 50 mm. In order to shorten the length of the optical path, the spacing between the signs is set to 10 mm at a ratio of 5 to 1 when calculating the RGB holograms of the real 3D scene. Then, the EEPMD-Net is trained after setting the benchmark distance to 300 mm and the layer spacing to 10 mm. Therefore, the recording distances of the “no parking”, “sidewalk”, “intersection” and “traffic light” signs are 300 mm, 310 mm, 320 mm, and 330 mm, respectively. The layer spacing of the holographic reconstructed image is determined by the training parameters of the EEPMD-Net. However, because the holographic reconstructed image also has focal depth, it is difficult for the camera to obtain a good focus or blurring effect when the layer spacing is small. In order to facilitate shooting, the layer spacing is set to 10 mm.

Meanwhile, the magnified images and the values of PSNR and SSIM of each sign are given. From the images and the values of PSNR and SSIM, it can be seen that the EEPMD-Net is able to realize high-quality holographic reconstruction, and the texture and layered details of the real 3D scene are well reconstructed. In addition, the proposed holographic camera can also be used for dynamic holographic 3D AR reconstruction, and the experimental results are shown in supplementary information S5.3 (Videos of the dynamic real scenes and holographic reconstruction are shown in videos 1 and 2).

## Discussion

Most currently reported holographic display systems focus more on realizing the recording and reconstruction of the virtual 3D objects. In this paper, we propose a holographic camera based on the liquid camera and the EEPMD-Net. The liquid camera to acquire the real 3D scene with true depth is demonstrated. In addition, the EEPMD-Net from the acquisition end of a real 3D scene to the output end of a high-fidelity hologram with true depth is proposed. The comparison of the performance parameters of the proposed holographic camera with other systems is shown in Table 2. The proposed EEPMD-Net can realize the training of real 3D scenes with more layers and different layer spacings, which can be achieved only by changing the number of layers and layer spacings in the model training parameters. There is still much work that can continue to be refined in future research. Currently, depth estimation techniques based on deep learning have become an important branch in the field of machine vision. Combining the depth estimation technique with the proposed EEPMD-Net is one of the directions to further improve the model performance. Moreover, it can also be attempted to further shorten the inference time of the model, which requires continued improvement of the network structure, including the adoption of new modules or the use of distillation techniques to reduce the number of parameters of the model.

**Table 2:** Comparison of characteristics between the proposed system and other systems

Methods	Hologram resolution	Response time	Calculation time	PSNR
Proposed	1920 × 1080	91 ms	≤53 ms	>25 dB
Yu et al.^10^	720 × 720	47 ms	48 ms	18 dB
Wang et al.^27^	1920 × 1080	>200 ms	>1000 ms	<15 dB

In this work, a holographic camera is proposed for acquiring the hologram of real 3D scene at true depth for the first time. The self-developed liquid camera, which is the core hardware of the holographic camera, is utilized to efficiently and accurately acquire images and the depth information of the real 3D scene. The core element of the liquid camera is an advanced electrowetting liquid lens with a large aperture of 10 mm. Then, the acquired scene information is input into the EEPMD-Net to realize the fast calculation of the hologram of the real 3D scene within 150 ms. Experimental results demonstrate that our proposed holographic camera is capable of realizing dynamic and low-noise hologram of the real 3D scene at true depth. The PSNR of the reconstructed image can reach ~28 dB. The proposed holographic camera is expected to be applied in the fields of 3D display, optical measurement, optical encryption, and so on.

## Materials and methods

### Training detail

The EEPMD-Net is built and trained with the PyTorch deep learning framework. The PyCharm integrated development tool is used as the platform for building the EEPMD-Net. To train and test the EEPMD-Net, a hybrid dataset consisting of the CREStereo dataset and the DIV2K dataset is used as the training dataset and validation dataset. The CREStereo dataset is a dataset obtained by Blender’s synthetic rendering technique, which contains complex scenes consisting of various objects with fine structures. Also, the CREStereo dataset provides accurate depth maps of these scenes. Since the CREStereo dataset is very large, 200 scenes are randomly selected from the CREStereo dataset as the training dataset and 50 scenes as the validation dataset. The DIV2K dataset contains 900 high-definition images, of which 800 images are used as the training dataset and 100 images are used as the validation dataset. The MiDaS depth estimation is used to obtain the depth maps for the DIV2K dataset and the Adam optimizer is used to train the EEPMD-Net. The hyperparameters in the optimizer are default values except for the learning rate. The training of the EEPMD-Net is divided into two stages. The first stage is the pre-training stage of the model, where the CREStereo dataset is used and the learning rate is 0.0004. The second stage is the fine-tuning stage of the model, and the DIV2K dataset is used in this stage with a learning rate of 0.0001. The number of training epochs for the first stage is 80 and the number of training epochs for the second stage is 50 with a mini-batch size of 1. The model is trained on a computer running Windows 11 operating system with an Intel Core i9-10980XE CPU and an NVIDIA GeForce RTX 3090 GPU (see supplementary information S5.4 for loss curves).

### Supplementary information

Supplementary information for Liquid lens based holographic camera for real 3D scene hologram acquisition using end-to-end physical model-driven network

Video 1

Video 2
