# Supplementary material for: Liquid lens based holographic camera for real 3D scene hologram acquisition using end-to-end physical model-driven network
Source: Light Sci Appl. 2024 Feb 29;13:62. doi: 10.1038/s41377-024-01410-8 (PMC10904790; doi:10.1038/s41377-024-01410-8)
Supplement: Supplementary file 1 — Supplementary information for Liquid lens based holographic camera for real 3D scene hologram acquisition using end-to-end physical model-driven network [file 41377_2024_1410_MOESM1_ESM.docx]

SUPPLEMENTARY INFORMATION

**Liquid lens based holographic camera for real 3D scene hologram acquisition using end-to-end physical model-driven network**

Di Wang^1,†^, Zhao-Song Li^1,†^, Yi Zheng^1^, You-Ran Zhao^1^, Chao Liu^1^, Jin-Bo Xu^1^, Yi-Wei Zheng^1^, Qian-Huang^1^, Chen-Liang Chang^2^, Da-Wei Zhang^2^, Song-Lin Zhuang^2^, and Qiong-Hua Wang^1,*^

^1^ *School of Instrumentation and Optoelectronic Engineering, Beihang University, Beijing 100191, China.*

^2^ *School of Optical-Electrical and Computer Engineering, University of Shanghai for Science and Technology, Shanghai, 200093, China.*

^†^*These authors contributed equally to this work.*

**Correspondence: QH Wang, E-mail: [qionghua@buaa.edu.cn](mailto:qionghua@buaa.edu.cn)*

14 pages, 13 figures, S1-S5

**S1: Optical structure of the liquid camera based on liquid lens**

The optical structure of the liquid camera based on liquid lens is shown in Fig. S1. The liquid lens consists of an upper electrode, a lower electrode, a conductive liquid, an insulating liquid, two pieces of window glass and a shell. The upper and lower electrodes and two pieces of window glass form a sealed space which is filled with two liquids. The upper electrode serves as a lens cavity and is sequentially coated with a dielectric layer and a hydrophobic layer on its inner surface. The lower electrode is directly connected to the conductive liquid and is well insulated from the upper electrode. The conductive liquid is at the bottom, while the insulating liquid is at the top, forming a naturally curved liquid-liquid interface between the two liquids.


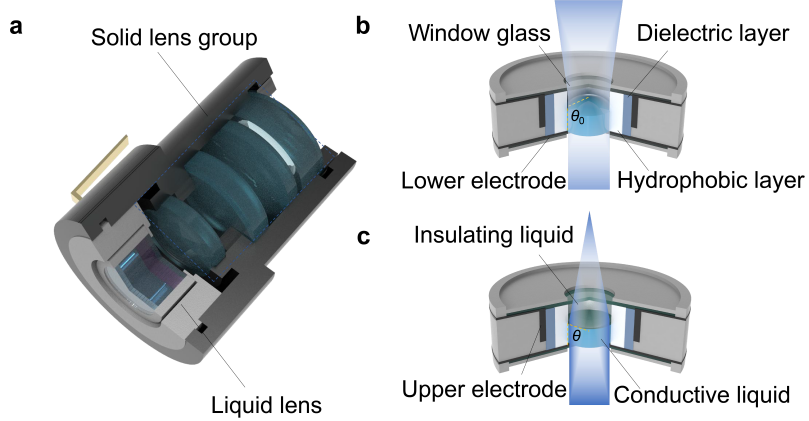


**Fig. S1 Optical structure of the liquid camera. a** Structure of the liquid camera. **b** Liquid lens working in concave lens state. **c** Liquid lens working in convex lens state.

**S2: Details of the EEPMD-Net**

**S2.1: Principle of the unsharp mask filter**

In order to enhance the edge information of the scene, an unsharp mask filter operation is applied in the EEPMD-Net. The basic idea of the unsharp mask filter is to strengthen the high frequency information connected with the detail and edge information. The unsharp mask filter operation has three steps. Firstly, the input image *I*(*x*, *y*) is multiplied with the Gaussian function to get a blurred image *I*_b_(*x*, *y*) of the input image.

$I_{\text{b}}\text{(}x,y\text{)}=I\text{(}x,y\text{)}\cdot\frac{1}{2\pi\sigma^{2}}\text{exp}\left[-\frac{x^{2}+y^{2}}{2\sigma^{2}}\right]$ (S1)

where *σ* is the standard deviation of the Gaussian distribution. Secondly, the blurred image *I*_b_(*x*, *y*) is subtracted from the input image *I*(*x*, *y*) to generate an unsharp mask *M*(*x*, *y*):

$M\text{(}x,y\text{)}=I\text{(}x,y\text{)}-I_{\text{b}}\text{(}x,y\text{)}$ (S2)

Finally, the unsharpened mask *M*(*x*, *y*) is subtracted from twice the input image *I*(*x*, *y*) to get the detail-enhanced output image *I*_o_(*x*, *y*):

$I_{\text{o}}\text{(}x,y\text{)=2}I\text{(}x,y\text{)}-I\text{(}x,y\text{)}\cdot\frac{1}{2\pi\sigma^{2}}\text{exp}\left[-\frac{x^{2}+y^{2}}{2\sigma^{2}}\right]$ (S3)

The Kornia library is used to construct the unsharp mask filters with a kernel size of 3×3 and a standard deviation *σ* of 1 for the Gaussian distribution.

**S2.2: Structures of network Ⅰ and network Ⅱ**

The structures of network Ⅰ and network Ⅱ are shown in Fig. S2. Network Ⅰ consists of four down-sampling blocks, five receptive field blocks, five up-sampling blocks, five parametric rectification linear unit (PReLU) function layers and a tangent hyperbolic (tanh) function layer. Network II consists of four down-sampling blocks, four receptive field blocks, four up-sampling blocks, four PReLU function layers and a tanh function layer. In order to optimize the performance of the model, the placement order of the down-sampling block, the receptive field block, and the up-sampling block is carefully designed (see supplementary material S2.3). When the image tensor is input to the down-sampling block, the channels of the image tensor are increased and the height and width of the image tensor are halved. The receptive field block is used to fully sense the features of the input image tensor, so the network can learn more information from the image tensor, which is beneficial to the generation of the high-fidelity holograms. When the image tensor is input to the up-sampling block, the channels of the image tensor are decreased and the height and width of the image tensor are doubled. The PReLU function layer is a transition layer, which is used to prevent over-fitting of the model and make the network train stably. By using the skip connections between the down-sampling block and the up-sampling block, gradient vanishing and network degradation can be avoided, so the network can be trained more efficiently.


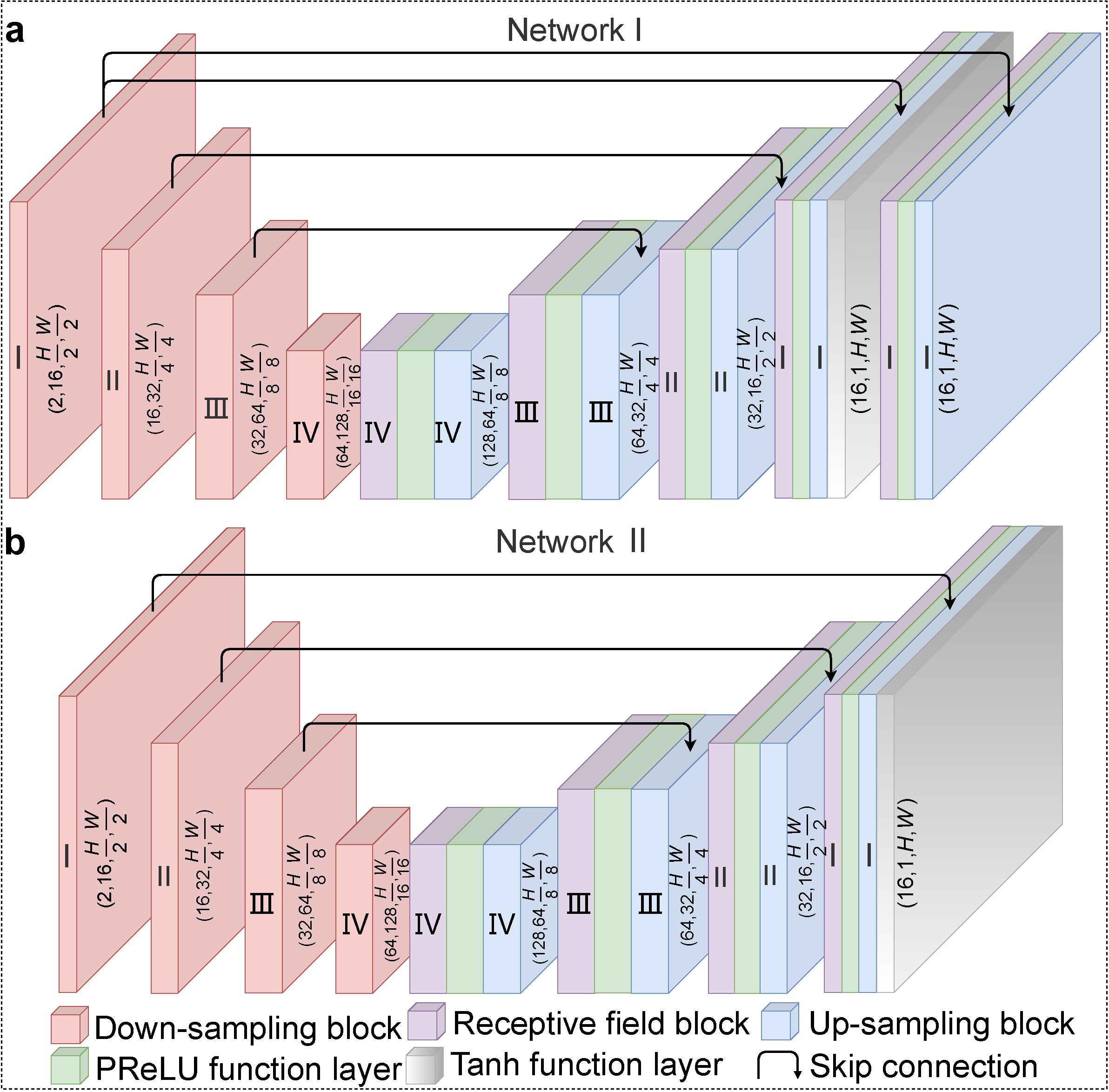


**Fig. S2 Detailed schematic structures of network I and network II of the EEPMD-Net. a** Structure of network Ⅰ. **b** Structure of network Ⅱ.

Fig. S3 shows the structures of the down-sampling block, up-sampling block, and receptive field block (supplementary material S2.4). Each down-sampling block consists of sequence nets I–III, and two 1×1 convolution kernels with a step size of 2 (conv2d(1, 2) in Fig. S3a). Each up-sampling block consists of sequence nets Ⅳ–Ⅵ and two 2×2 transposed convolution kernels with a step size of 2 (transposed conv2d(2, 2) in Fig. S3b). Each receptive field block consists of branches I–IV, two conv2d(1, 1), and a concatenation operation. “×0.2” in Fig. S3c represents that each element of the input tensor is multiplied by 0.2.

The structures of sequential nets I–VI and branches I–IV are shown in Figs. S3d–m. In the EEPMD-Net, normalization methods such as batch normalization are not used to prevent the network from ignoring the features of the input image tensor. As shown in Figs. S3g–i, the up-sampling block of the EEPMD-Net contains a composite convolution algorithm consisting of transposed convolution, pixel shuffle and bicubic interpolation, in order to avoid the checkerboard effect when only transposed convolution is used for up-sampling and realize the generation of holograms with low noise. As shown in Figs. S3j–m, the dilated convolution (for example, conv2d(3,1,5) represents 3×3 convolution with a step size of 1 and a dilation rate of 5) is used in the receptive field block. By combining the dilated convolutions with different dilation rates, the receptive field block has the ability to fully extract various features in the image tensor, which is helpful to generate the high-fidelity holograms. The receptive field sizes of branches I-IV of the receptive field block are 3, 7, 9, and 13.


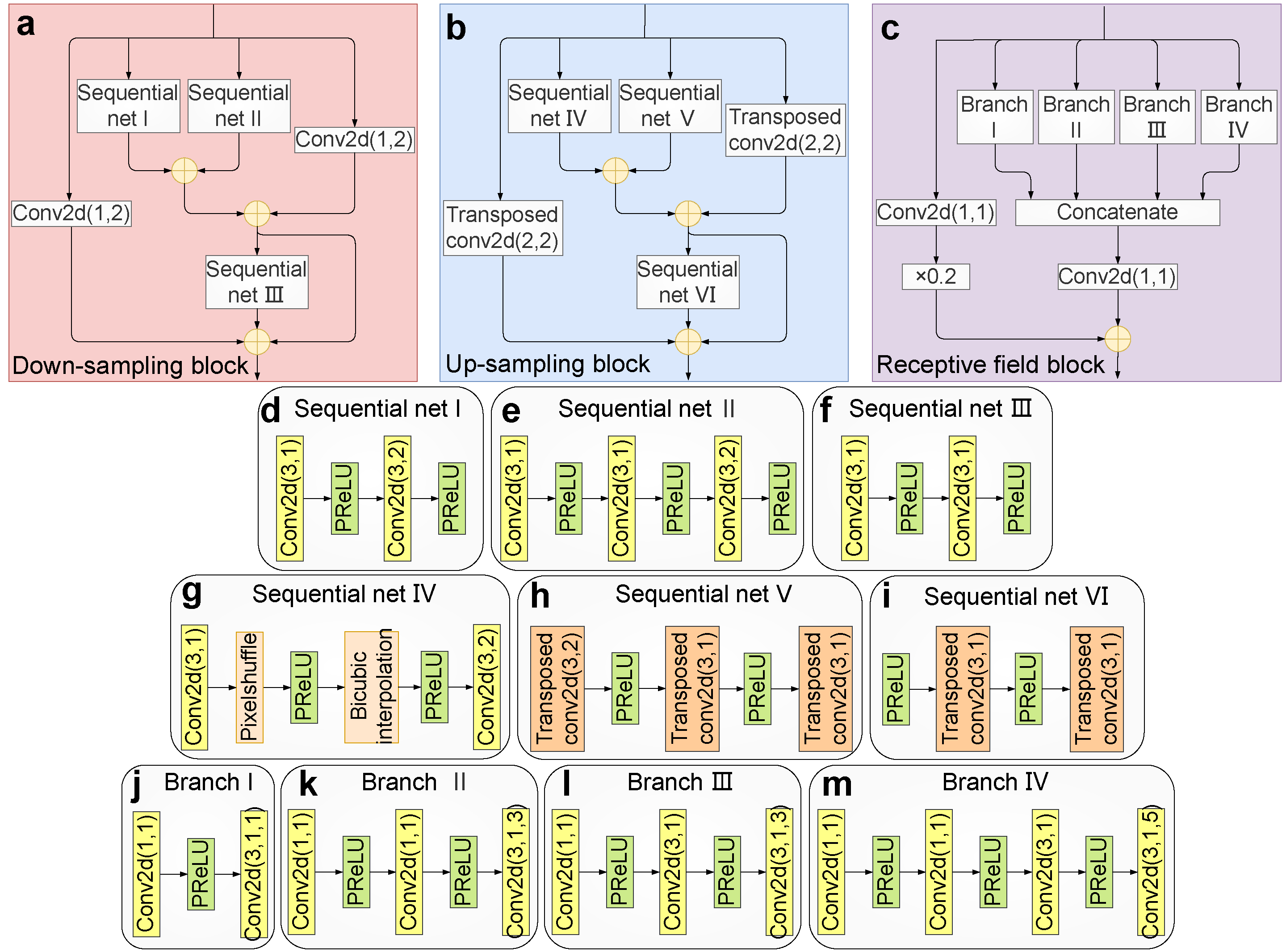


**Fig. S3 Structures of the down-sampling block, up-sampling block, and receptive field block. a** Structure of the down-sampling block. **b** Structure of the up-sampling block. **c** Structure of the receptive field block. **d–i** Structures of sequential nets I–Ⅵ. **j–m** Structures of branches I–IV.

**S2.3: Explanations of the down-sampling block, up-sampling block and receptive field block**

The down-sampling block is used to reduce the height and width of the image tensor and increase the number of channels. For example, as shown in Fig. S2a, down-sampling block I is labeled (2, 16, *H*/2, *W*/2), which means that when the image tensor passes through down-sampling block I, the number of channels of the image tensor changes from 2 to 16, and the height and width of the image tensor change from *H* and *W* to *H*/2 and *W*/2, respectively. The up-sampling block is used to increase the height and width of the image tensor and decrease the number of channels. For example, as shown in Fig. S2a, up-sampling block II is labeled (32, 16, *H*/2, *W*/2), which means that when the image passes through up-sampling block II, the number of channels of the image tensor changes from 32 to 16, and the height and width of the image tensor change from *H*/4 and *W*/4 to *H*/2 and *W*/2, respectively. Through the skip connection, the output of down-sampling block III is added to the output of up-sampling block III as the input of receptive field block (RFB) II, as shown in Fig. S2b.

The RFB is used to expand the feature recognition ability of the network for the input tensor. In the EEPMD-Net, the RFB is inserted after the up-sampling block. This is designed to pass as much information as possible after the up-sampling block to the back end of the network. The related experimental results have confirmed the effectiveness of our proposed network for preserving image details. Moreover, several other different network structures are tested to ensure that the location where the RFB is located in the proposed EEPMD-Net is the optimal location. The test is performed by comparing the rate of decline of the training loss for different network architectures within the same number of epochs with the same training parameters and dataset. It is considered that the faster the training loss decreases, the better the network structure is. The training loss curves obtained by the test are shown in Fig. S4.

As shown in Fig. S4, the network is trained for 50 epochs, the learning rate is set to 0.0004, and the CREStereo dataset is used as the training and validation dataset. When there is no RFB, the values of training loss and validation loss of the network are much higher than in other cases, which proves that the inserted RFB can speed up the convergence of the network. When the RFBs are inserted into the up-sampling and down-sampling blocks, the values of training loss and validation loss of the network are higher. This indicates that the performance of the network is affected by the location of RFBs, and the performance of the network is not improved by the increase in the number of RFBs. It is worth noting that although the training loss value of the network with the RFB after the down-sampling block is low at the beginning of 10 epochs. However, after 50 epochs, the training loss value and the validation loss value of the network with RFB after the down-sampling block are close to the training loss value and validation loss value of the network with RFB after the up-sampling block. A closer look also reveals that after 50 epochs, the training loss and validation loss values of the network with RFB after the up-sampling block are lower. The reason is that, in terms of the trend of the loss curve, the gradient of the loss value change of the network with RFB after the up-sampling block is higher than that of the network with RFB after the down-sampling block, which is more favorable for the network to converge to a lower loss value. Therefore, the position of the RFB in the network is finally set after the up-sampling block.


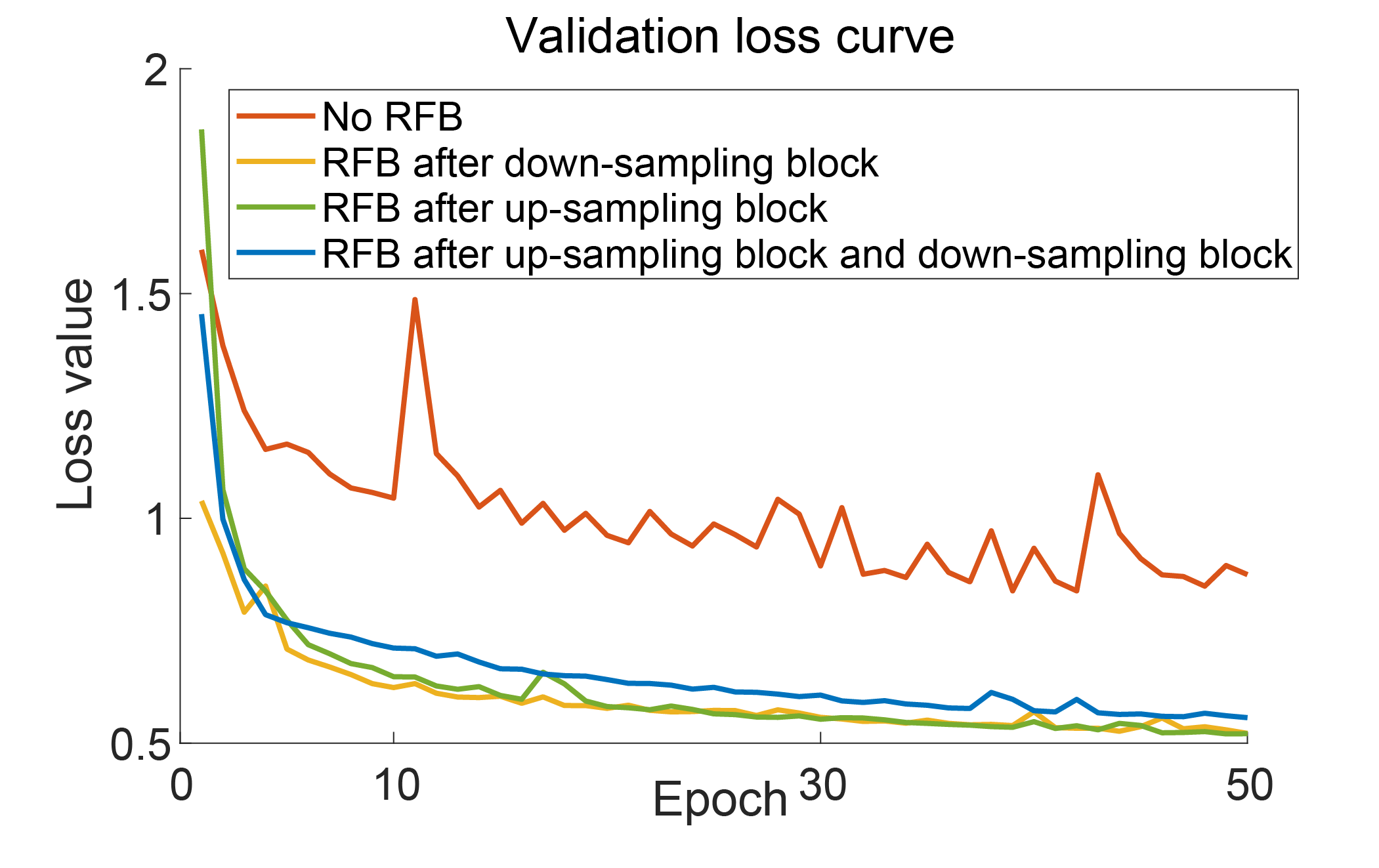


**b**


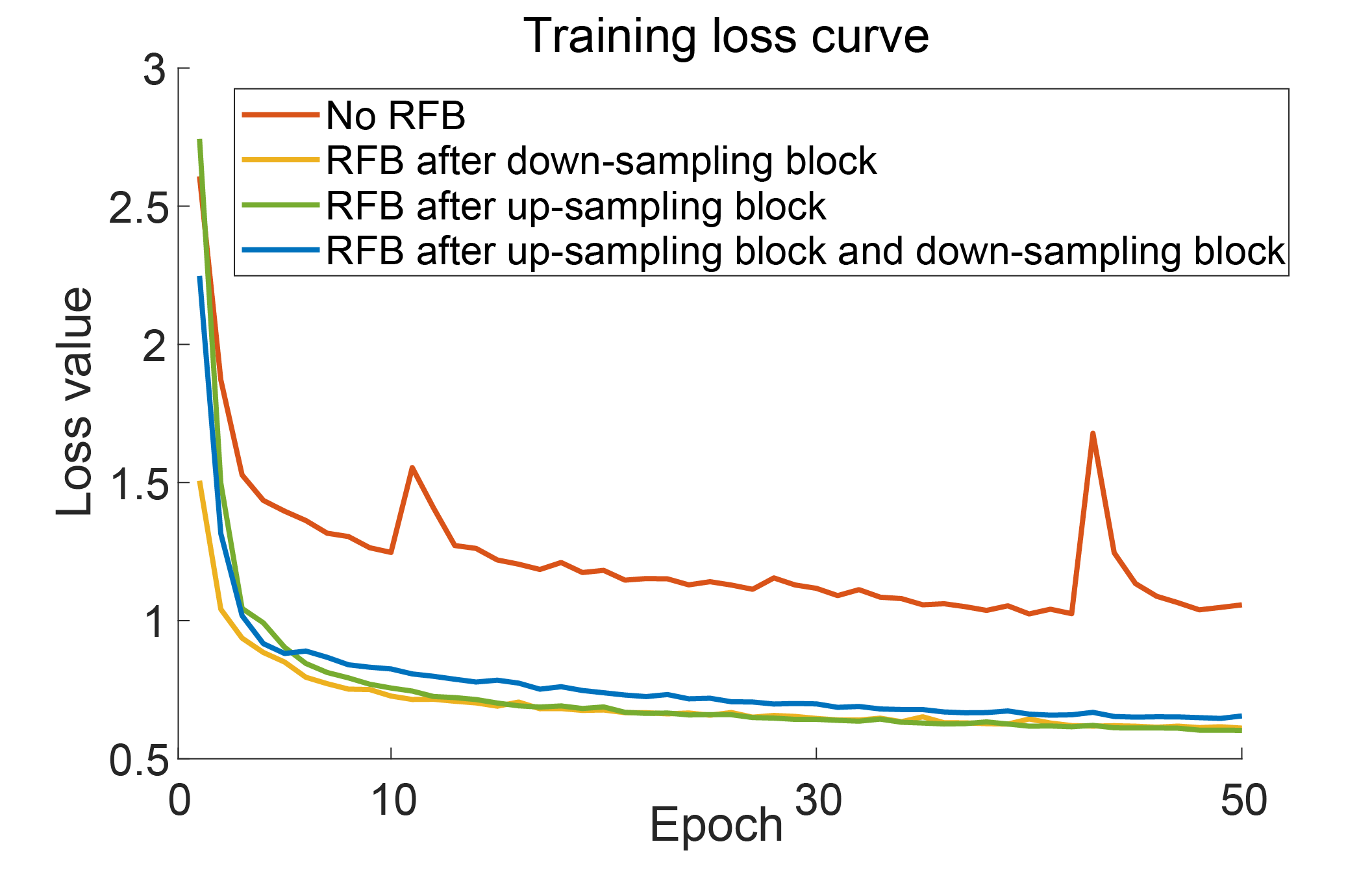


**a**

**Fig. S4 Loss curves for different network structures. a** Training loss curve. **b** Validation loss curve.

**S2.4: Additional description of the structures of the down-sampling block, up-sampling block, and RFB**

As shown in Fig. S3a, after the image tensor enters the down-sampling block, it is merged after passing through sequential net I, sequential net II, and a conv2d(1, 2), respectively. Then, the merged data are output after passing through sequential net III and a skip connection. Meanwhile, the output of the down-sampling block consists of the image tensor directly passing through conv2d (1, 2). As shown in Fig. S3b, after the image tensor enters the up-sampling block, it is merged after passing through sequential net IV, sequential net V, and a transposed conv2d(2, 2), respectively. Then, the merged data are output after passing through sequential net VI and a skip connection. Meanwhile, the output of the up-sampling block includes the data of the image tensor directly passing through a transposed conv2d(2, 2). As shown in Fig. S3c, the image tensor enters the RFB and passes through branches I–IV. The output data of each branch is concatenated in the channel dimension. The concatenated data is output after passing through a conv2d(1, 1). Meanwhile, the output of the RFB also includes the data of the image tensor passed through a conv2d(1, 1) and then ×0.2.

In the EEPMD-Net, normalization methods such as batch normalization (BatchNorm) and instance normalization (InstanceNorm), which are common in other deep learning-based hologram calculation methods, are not used. The reason is that while BatchNorm and InstanceNorm have the effect of preventing gradient vanishing and controlling gradient explosion, the normalization methods converge the statistical distributions of each layer of the network. It would cause the trained network to ignore the differences between the features (e.g., color, contrast, brightness, etc.) of the input image, thus affecting the quality of the hologram output by the network. Meanwhile, BatchNorm and InstanceNorm will also affect the convergence speed of the network and occupy the memory resources of the computer. Therefore, in order to improve the performance of the network, the normalization methods such as BatchNorm and InstanceNorm are not used in the EEPMD-Net.

The PReLU is a variant of the rectified linear unit (ReLU) that assigns a tiny gradient (usually a small number between 0 and 1) to the part of the input image tensor that is smaller than 0, thus solving the problem of gradient direction jaggedness that may occur when using the ReLU. The transposed convolution is a widely used up-sampling method, and using only the transposed convolution for up-sampling produces the checkerboard effect due to the problem of uneven overlap. In order to avoid the checkerboard effect while maintaining the calculation speed of the network, the up-sampling block of the EEPMD-Net is composed of not only the transposed convolution but also pixel shuffle and bicubic interpolation.

Compared with normal convolution, the dilated convolution introduces a hyperparameter called dilatation rate, which is used to define the distance between adjacent elements of the convolution. The larger the dilatation rate is, the larger the receptive field of the dilated convolution is. Thus, the dilated convolution makes branches I-IV in Fig. S3c have different receptive scales for the input image tensor.

**S2.5: Description of the band-limited angular spectrum method**

In the EEPMD-Net, the complex amplitude distribution of the SLM amplitude and phase fields *U*_SLM_ (*x*, *y*) can be expressed as follows:

 (S4)

where *U*_o_ (*x*, *y*) is the complex amplitude distribution of the target amplitude and phase fields, *j* represents the imaginary number, F{ } and F^-1^{ } represent the Fourier transform and inverse Fourier transform, respectively. *λ*_n_ (*n*=R, G, B) represents the wavelength of the coherence light, and *z*_0_ represents the benchmark recording distance. *f*_x_ and *f*_y_ represent the horizontal and vertical coordinates of the frequency domain, respectively.

*w*(*f*_x_, *f*_y_) is a frequency domain filter used to impose frequency domain constraints and it can be expressed as follows:

$w(f_{\text{x}},f_{\text{y}})=f_{\text{x}}<[(\frac{2z\cdot\lambda_{\text{n}}}{M\cdot p})^{2}+\lambda^{2}]^{-\frac{1}{2}}\text{ and }f_{y}<[(\frac{2z\cdot\lambda_{\text{n}}}{N\cdot p})^{2}+\lambda^{2}]^{-\frac{1}{2}}$ (S5)

where *z* represents the diffraction distance, *p* represents the pixel pitch, *M* and *N* represent the horizontal and vertical resolution of the SLM, respectively.

When the RGB holograms are calculated, the intensity of the reconstructed images can be expressed as:

 (S6)

where ||∙|| means taking the modulus of the expression in the symbol, *I*_i_ (*x*, *y*) represents the intensity of the reconstructed image of the *i* th layer, *U*_H_ (*x*, *y*) represents the complex amplitude distribution of the hologram, and ∆*z* represents the spacing between the layers of the real 3D scene.

**S2.6: Description of the loss functions of the EEPMD-Net**

The mean square error (MSE) loss is a common loss function in image generation tasks for deep learning, which optimizes the peak signal-to-noise ratio (PSNR) by calculating the difference in pixel values between the generated image and the target image. Even if the PSNR of the generated image can be optimized to a higher value, the generated image loses details and texture information due to smoothing caused by the MSE loss. In order to make the generated images visually closer to the target images recognized by the human eye, the loss function of the EEPMD-Net is constructed by combining the perceptual (PE) loss, the multi-scale structural similarity (MS-SSIM) loss, and the total variance (TV) loss, and the MSE loss. The basic idea of the PE loss is to utilize a pre-trained convolutional neural network (usually VGG16 or VGG19) to extract high-level features of the target image. Then, the difference in pixel values between the generated image and the target image is calculated in the feature space. Compared with the MSE loss, the PE loss can better preserve the details and textures of the target image in the generated image, thus avoiding blurring or distortion of the generated image. In the EEPMD-Net, a pre-trained VGG19 is used to calculate the PE loss, and the PE loss can be expressed as follows:

$Loss_{\mathrm{PE}}=\sum_{\text{k}} \frac{1}{C_{\mathrm{k}}\times H_{\mathrm{k}}\times W_{\mathrm{k}}}\left\|\text{Ψ}_{\mathrm{k}}(s\times\text{Θ})-\text{Ψ}_{\mathrm{k}}(\hat{\text{Θ}})\right\|_{2}^{2}$ (S7)

where ||∙||2 2 represents to calculate the square of the L2 norm of the expression within the symbol, *Ψ*_k_ represents the *k* th convolutional layer used for feature extraction in the VGG19, the number *k* of the convolutional layer is ‘2, 4, 6, 10, 14’, *s* is a coefficient with the value of 0.95, *Θ* represents the intensity of the complete reconstructed image, $\hat{\text{Θ}}$ represents the intensity of the real 3D scene, *C*_k_×*H*_k_×*W*_k_ represents the channel×height×width of the *k*th convolutional layer.

In the EEPMD-Net, the MS-SSIM loss is used to measure the structural similarity between the complete reconstructed image and the enhanced 3D scene at different scales. Compared with the MSE loss, the MS-SSIM loss takes into account the visual experience of the human eye, and the formula of the MS-SSIM loss is expressed as follows:

$Loss_{\mathrm{MS}}=1-\prod_{m=1}^{M} (\frac{2\mu_{\text{g}}\mu_{\text{t}}+c_{1}}{\mu_{\text{g}}^{2}+\mu_{\text{t}}^{2}+c_{1}})^{\beta_{\text{m}}}(\frac{2\sigma_{\text{gt}}+c_{2}}{\sigma_{\text{g}}^{2}+\sigma_{\text{t}}^{2}+c_{2}})^{\gamma_{\text{m}}}$ (S8)

where *m* is a scaling factor, and the height and width of the input image are scaled by a factor of 2^m-1^ (*m*=1, ..., *M*), *μ*_g_ and *μ*_t_ represent the mean values of the generated and the target image, respectively, *σ*_g_ and *σ*_t_ represent the standard deviations of the generated and the target image, respectively, *σ*_gt_ represents the covariance of the generated and the target image, *c*_1_ and *c*_2_ are two constant terms that are used to prevent the denominator from being zero, *β*_m_ and *γ*_m_ stand for the relative importance of the mean and variance terms.

The TV loss makes the generated image visually closer to the target image. The basic idea of the TV loss is to utilize the total variance of the image as a measure of the smoothness of the image, thus suppressing noise and artifacts. In the EEPMD-Net, the TV loss is used as the regularizer. The TV loss is expressed as follows:

$Loss_{\text{TV}}=\sum_{u,v} \sqrt{(p_{\text{u,v-1}}-p_{\text{u,v}})^{2}+(p_{\text{u+1,v}}-p_{\text{u,v}})^{2}}$ (S9)

where *p*_u,v_ represents a pixel point of the input image. The TV loss calculates the square root of the sum of the squares of the differences between each pixel point *p*_u,v_ and the neighboring pixels *p*_u,v-1_ in the horizontal direction and *p*_u+1,v_ in the vertical direction, respectively. The TV loss is obtained by summing up all the pixel points after completing the calculation.

Meanwhile, in order to ensure the normalization of the SLM amplitude field at different wavelengths, the SLM amplitude field needs to be constrained. In the EEPMD-Net, the constraint of the SLM amplitude field is realized by the MSE loss between the mean value of the SLM amplitude field $\overline{O}_{\text{SLM}}$ and the SLM amplitude field *O*_SLM_. In summary, the loss function of the EEPMD-Net can be expressed as follows:

$Loss=\alpha Loss_{\mathrm{PE}}+\beta Loss{}_{\text{MS}}+\eta Loss_{\text{TV}}+\left\|\overline{O}_{\text{SLM}}-O_{\text{SLM}}\right\|_{2}^{2}$ (S10)

where *ɑ*, *β*, and *η* are the coefficients of the PE loss, the MS-SSIM loss and the TV loss, respectively. In the EEPMD-Net, *ɑ*, *β*, and *η* are set to 0.05, 1, and 1×10^-5^, respectively.

**S3: Additional details on the electrowetting-based liquid lens**

**S3.1: Transmission efficiency of the liquid lens**

The transmission efficiency of the fabricated liquid lens is measured with a spectrometer (Type of Aurera 4000, CNI co., Ltd., China), as shown in Fig. S5.


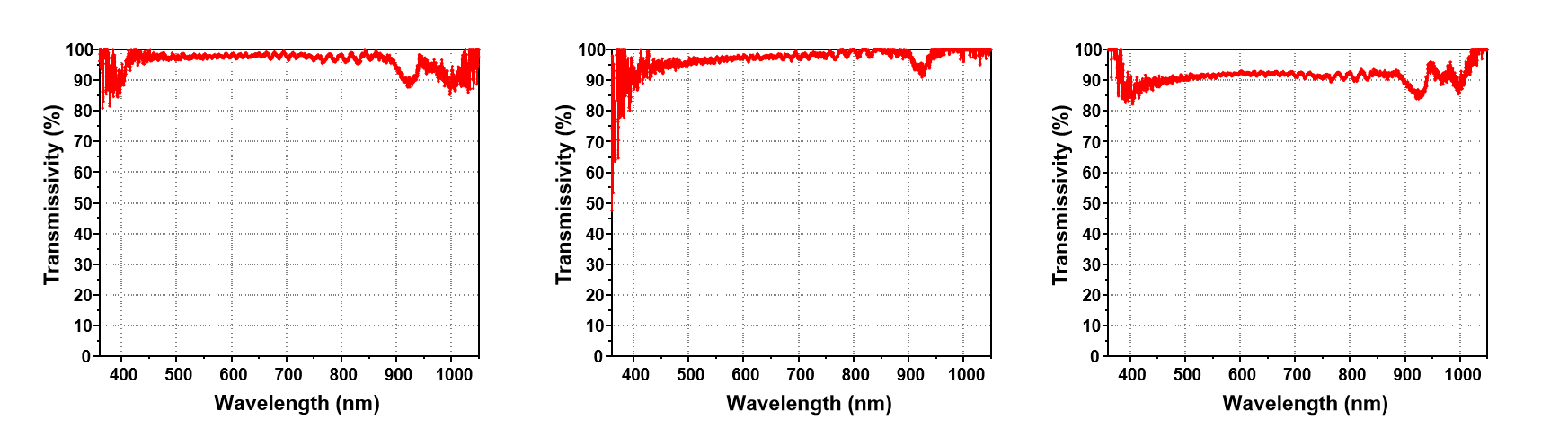


**a**

**b**

**c**

**Fig. S5 Transmission efficiency of the liquid lens.** **a** Transmittance of the conductive liquid. **b** Transmittance of the insulating liquid. **c** Total transmittance of the liquid lens.

**S3.2 Testing methods of the response time and optical power of the liquid lens**

The response time of the fabricated liquid lens is closely related to the performance of the system. The testing system is assembled using a 632.8 nm helium-neon laser, a Si-amplified detector and an oscilloscope. The Si-amplified detector converts optical signals into voltage signals, which are then displayed on the oscilloscope. We test the response time of the liquid lens from no voltage applied to the application of a 100 V, 1 kHz sine wave voltage signal. The voltage signal displayed on the oscilloscope is normalized, and the time taken from 10% to 90% of the maximum voltage is considered as the response time of the fabricated lens.

The optical power of the fabricated liquid lens is tested by using an optical power measurement device, and applying voltages ranging from 0 V to 100 V to the liquid lens. The initial optical power of the liquid lens is -5 m^-1^, with a threshold voltage of 10 V. When a voltage of 30 V is applied, the lens optical power becomes 0, and the liquid-liquid interface is planar. At 40 V, the lens optical power is 3.22 m^-1^, and when 100 V is applied, the lens optical power reaches 7.03 m^-1^.

**S3.3: Imaging performance calibration of the liquid camera**

Before capturing and measuring the depth of the real scene, calibrating the resolution of the liquid camera with different depths and voltages through experiments can provide a more reliable basis for our depth measurement. The liquid camera is used to capture a resolution test target USAF 1951 held on a precision mobile platform. We move the resolution test target within a depth range from 10 cm to 25 cm in the step of 1 cm and capture the images with different voltages. Some captured images are shown in Fig. S6. As can be seen, the liquid camera has a maximum resolution of 14.25 lp/mm when capturing objects 10 cm away, and a maximum resolution of 5.66 lp/mm when capturing objects 25 cm away. In addition, when capturing objects at a certain depth, the depth of field variation can be clearly seen as the driving voltage changes. Therefore, we can measure the depths of the objects based on the trend of image clarity change with different voltages. In this paper, the longitudinal resolution (or depth measurement accuracy) is limited to 1 cm within the depth range from 10 cm to 25 cm, which is determined by the designed depth calibration step. A smaller depth calibration step or more advanced image clarity measurement algorithms will contribute to more accurate depth measurement.


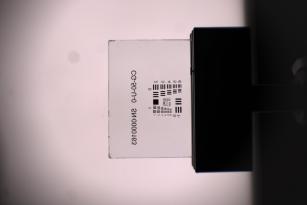

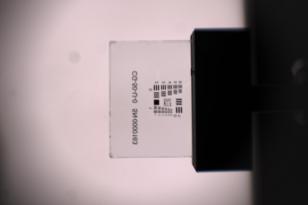

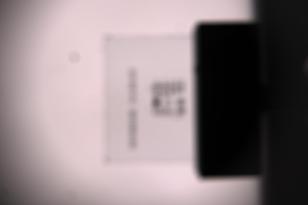

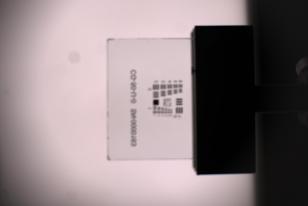

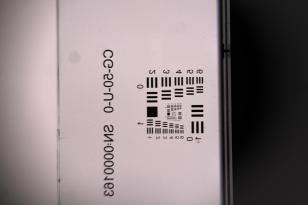

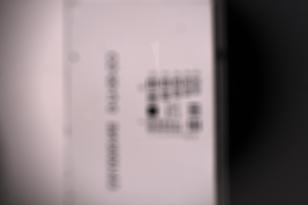

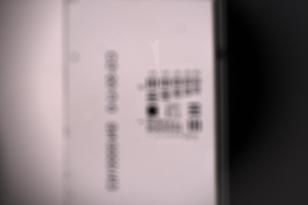

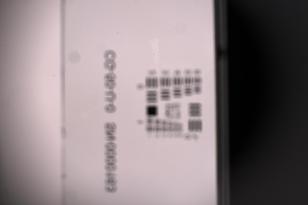

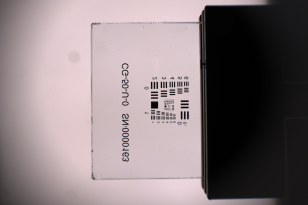

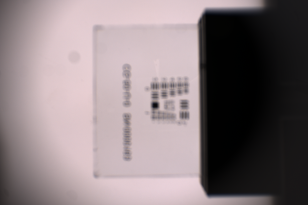

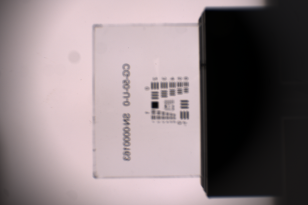

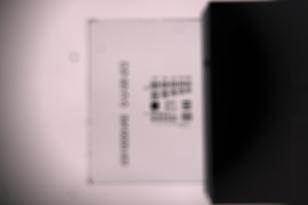

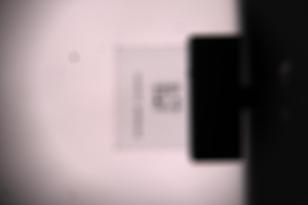

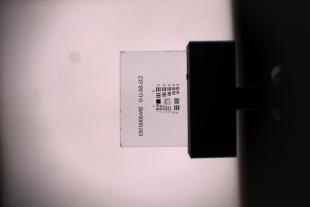

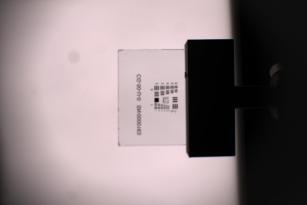

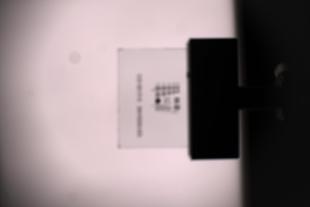


10 cm, 14 V

10 cm, 18 V

10 cm, 23 V

10 cm, 32 V

15 cm, 14 V

15 cm, 18 V

15 cm, 23 V

15 cm, 32 V

20 cm, 14 V

20 cm, 18 V

20 cm, 23 V

20 cm, 32 V

25 cm, 14 V

25 cm, 18 V

25 cm, 23 V

25 cm, 32 V

**Fig. S6 Resolution calibration of the liquid camera with different depths and voltages.**

It can also be observed that the angle of view changes slightly when changing the driving voltages, which is called the lens focus breathing effect. We calibrate this change and record the magnification correction factor curve, as shown in Fig. S7. By zooming in and cropping the captured images based on the curve, a consistent captured angle of view can be achieved. In this way, the region of interest will not drift with changes in voltage, ensuring the calculation accuracy of clarity variation trend when capturing the target object.


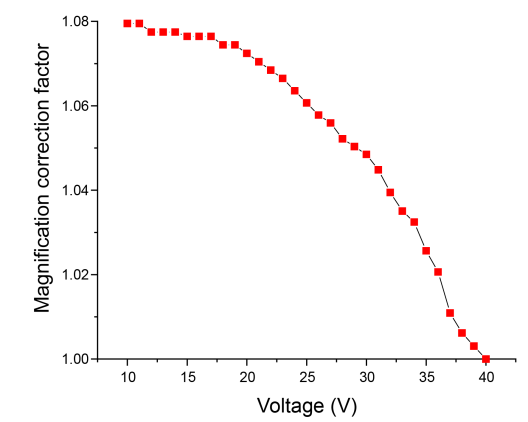


**Fig. S7 Magnification calibration of the liquid camera with different voltages.**

**S3.4: Resolution of the liquid camera**

The resolution of the liquid camera is shown in Fig. S8. It can be found that the focusing range and depth of field of the liquid camera change with the driving voltage. The focusing range of the liquid camera can fully cover from 10 cm to 25 cm when the driving voltage is between 10 V and 40 V.


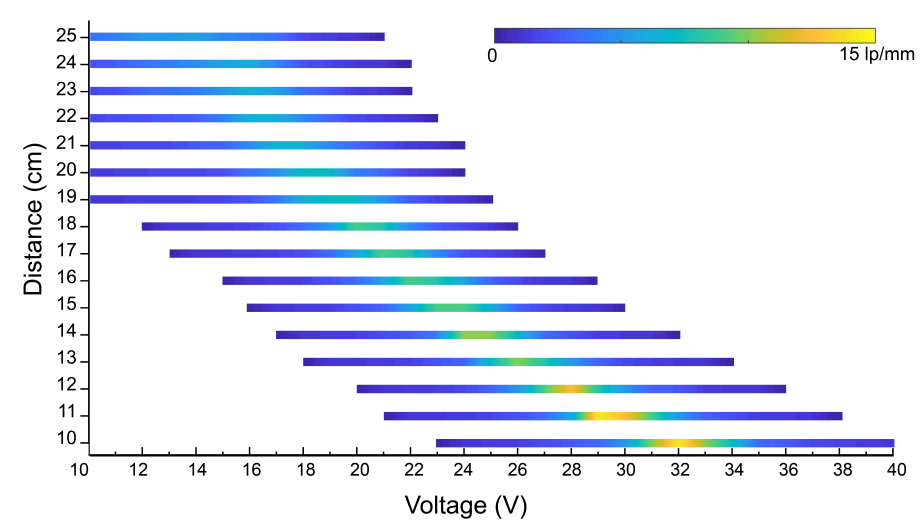


**Fig. S8 Resolution of the liquid camera.**

**S4: Description of the image capture and depth measurement**

**S4.1: Mask generation method in the depth measurement**

In order to improve the universality of the depth measurement algorithm, the regions of interest set in the experiments are all square. Therefore, it is also necessary to extract the complete shapes of the target objects with corresponding depths. In this paper, we design a mask generation method to achieve this function. The process is shown in Fig. S9. Firstly, the defocus estimation^1^ is executed. The edge positions of the objects are extracted after performing Gaussian filtering with a standard deviation of *σ*_1_ and Canny edge detection. Then the gradient amplitude ratio *R* in the edge position is calculated which can be defined as follows:

$R=\sqrt{\frac{\nabla i_{1x}^{2}+\nabla i_{1y}^{2}}{\nabla i_{2x}^{2}+\nabla i_{2y}^{2}}}$ (S11)

where ∇*i*_1_*_x_* and ∇*i*_1_*_y_* are the gradients along the *x* and *y* directions of the image after performing Gaussian filtering with the standard deviation of *σ*_1_, respectively. ∇*i*_2_*_x_* and ∇*i*_2_*_y_* are the gradients along the *x* and *y* directions of the image after performing Gaussian filtering with the standard deviation of *σ*_2_ respectively. For the edge of an object with a smaller degree of defocusing, the impact of secondary blur on it is greater, which means that *R* is greater. Therefore, *R* can reflect the degree of defocusing of the object. Then the defocus estimation value *σ* can be expressed as:

$\sigma\text{=}\sqrt{\frac{R^{2}\sigma_{1}^{2}-\sigma_{2}^{2}}{1-R^{2}}}$ (S12)

When calculating Eq. (S12), if there is an imaginary number term, it can be directly set to zero, and it is also recommended to set the upper threshold to reduce the impact of noise. After applying the matting Laplacian^2^ to perform the defocus map interpolation, the complete defocus estimation map *d* can be acquired:

$(L+\lambda D)d=\lambda Dd'$ (S13)

where *L* is the Laplace matrix, *D* is the diagonal matrix, λ is the constraint constant, and *d’* is the sparse defocus estimation map.

After binarizing the complete defocus estimation maps, we extract the main shapes of the focused target objects, but some of them are still incomplete, such as the “traffic light” sign. Then, we perform morphological processing on the binarized defocus estimation maps. The erosion operation is performed on the defocus estimations map to further remove noise, and then the dilation operation is performed. Finally, the complete masks of the target objects are generated.


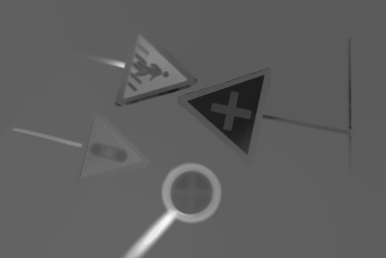

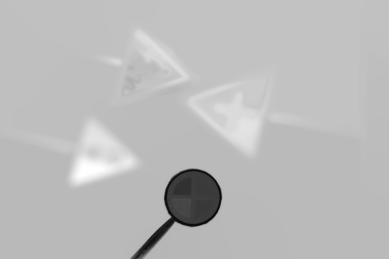

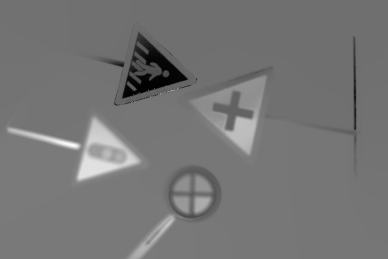

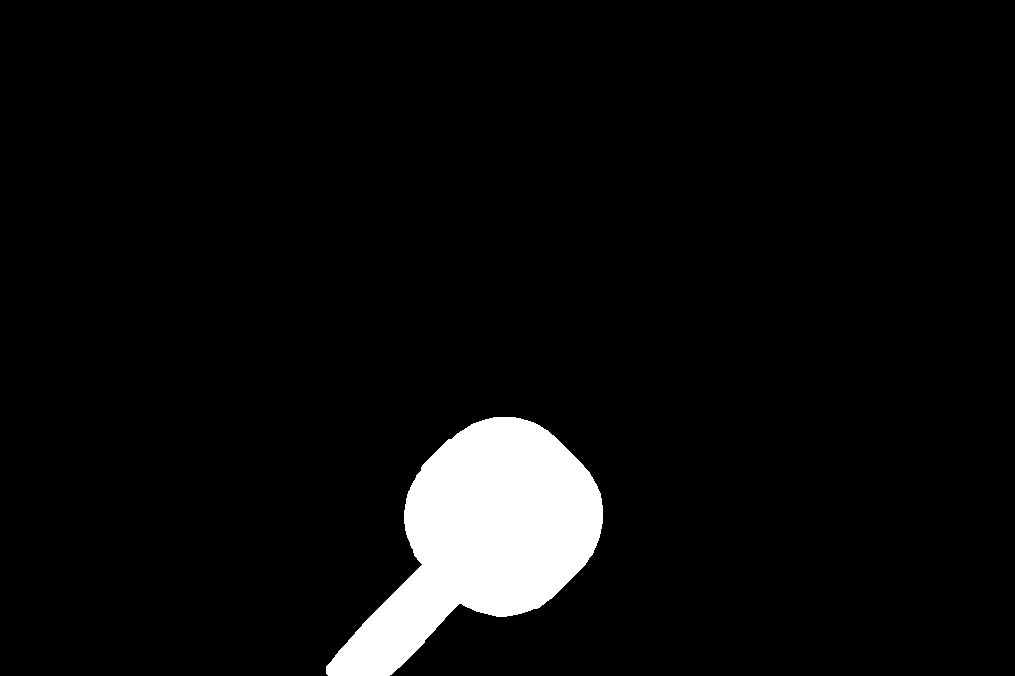

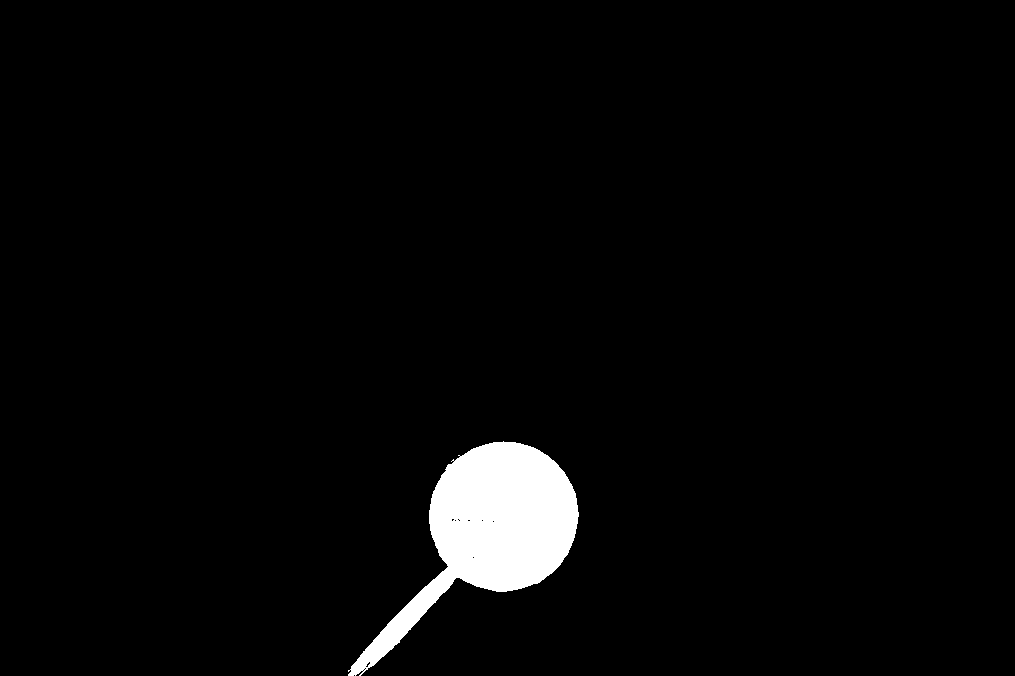

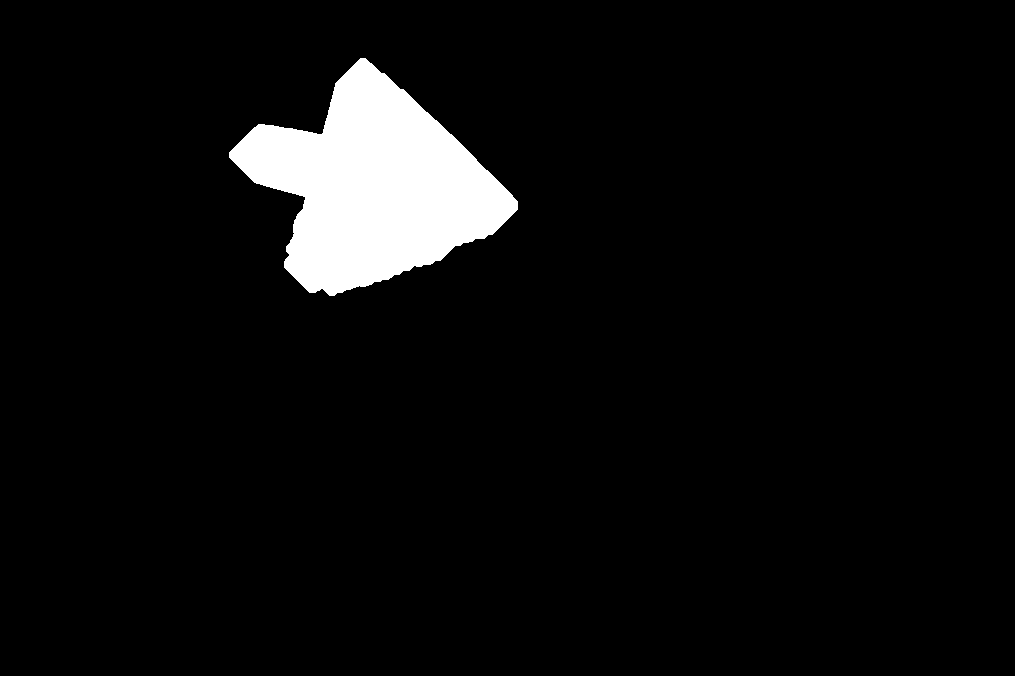

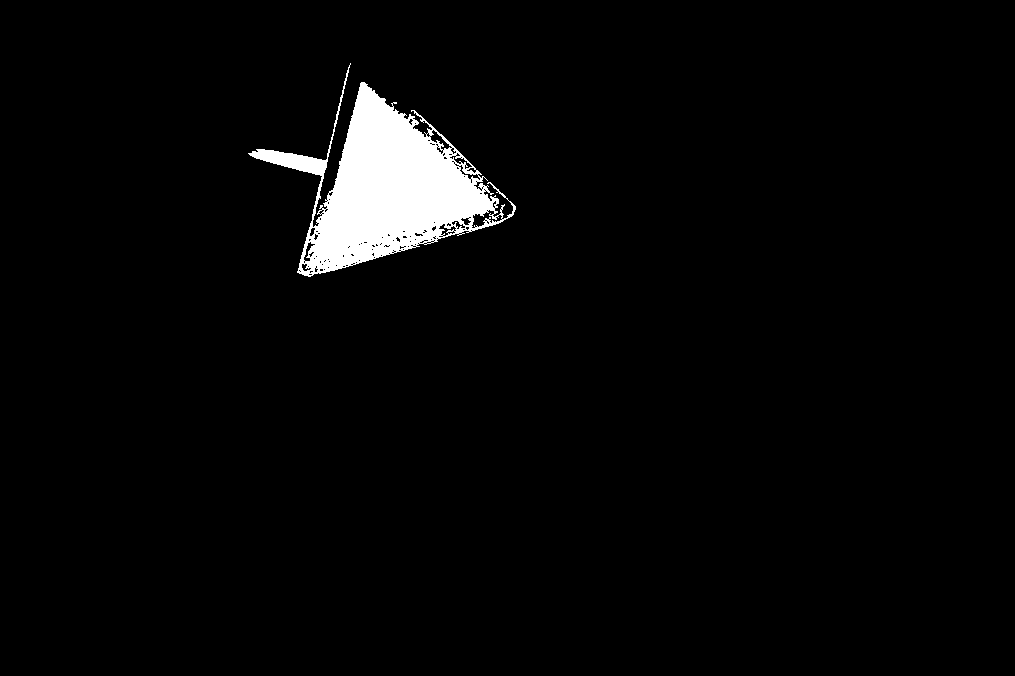

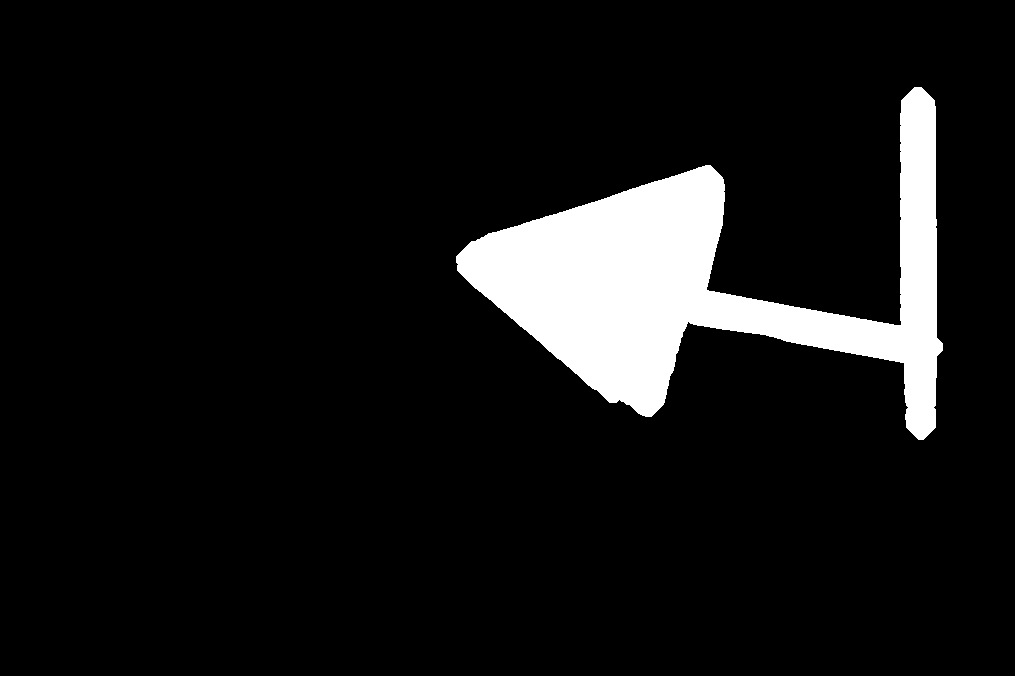

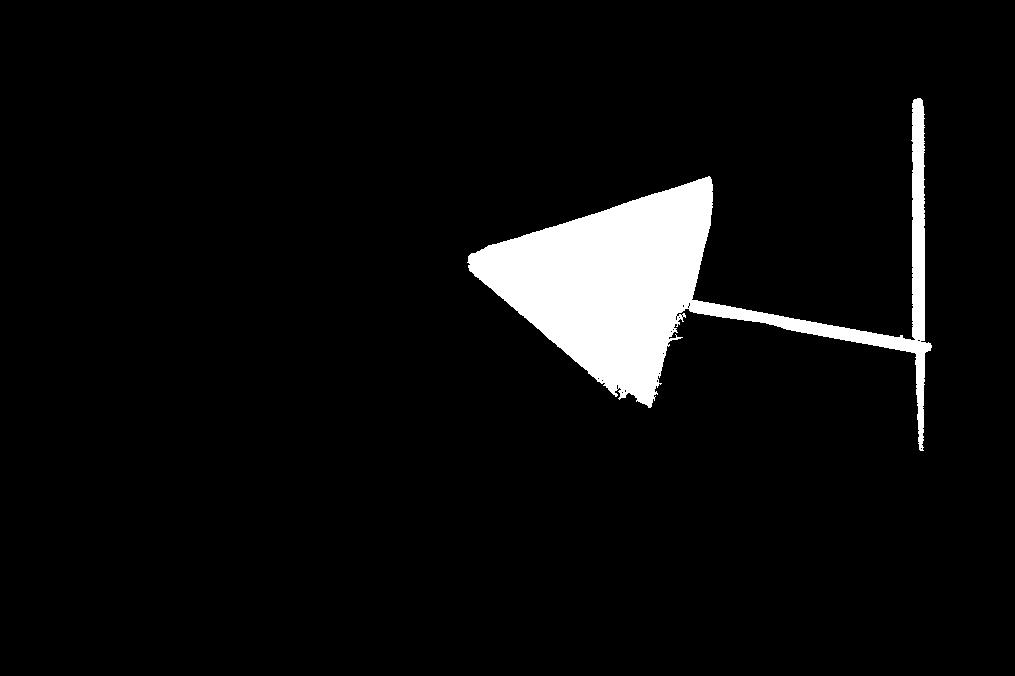


**Defocus**

**estimation**

**Binarization**

**processing**

**Morphological**

**processing**

**Fig. S9 Mask generation process of the focused region in the captured images.**

**S4.2: Results of the scene fusion and depth measurement**

After extracting the complete shape of each target sign based on the generated masks, a fused scene with all the focused target signs and depth map is generated according to the set number of layers, as shown in Fig. S10. The fused scene and depth map are then input into the neural network for holographic calculation.


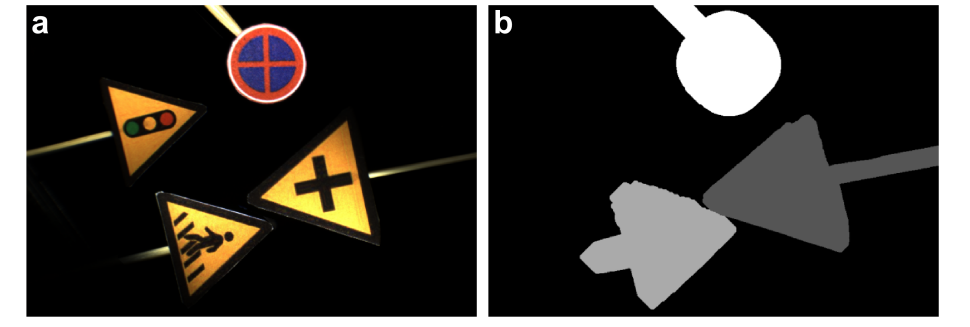


**Fig. S10 Scene fusion and depth measurement. a** Fused scene in which all target signs are focused. **b** Depth map of the fused scene.

**S5: Additional description of the experiment**

**S5.1: Comparison of the calculation time among the ED method, DP method, SGD method and EEPMD-Net.**

The calculation time of the RGB holograms of the “castle” using the ED method is 2609 ms, 2767 ms and 2538 ms, respectively. The calculation time for the RGB holograms using the DP method is 2663 ms, 2796 ms and 2820 ms, respectively. The calculation time of the RGB holograms by using the SGD method is 78 s. The calculation time of the RGB holograms using the EEPMD-Net is 51 ms, 53 ms and 51 ms, respectively. It can be found that the calculation speed of the EEPMD-Net is more than 40 times faster than that of the DP and EP methods, and more than 400 times faster than that of the SGD method, which is enough to prove the advantage of the EEPMD-Net in terms of calculation time.

**S5.2: Evaluation metrics for the quality of the reconstructed image**

The PSNR and structural similarity^3^ (SSIM) are used to evaluate the quality of the reconstructed image. The PSNR can be expressed as follows:

$\text{PSNR}=20\cdot\lg\left(\frac{1}{\sqrt{\frac{1}{mn}\sum_{i=0}^{m-1} \sum_{j=0}^{n-1} \left\|X(i,j)-Y(i,j)\right\|^{2}}}\right)$ (S14)

where *X*(*i*, *j*) and *Y*(*i*, *j*) represent the object image and the reconstructed image with a size of *m*×*n*, respectively. The pixel values of the calculated image are taken in the range of 0 to 1. The SSIM can be expressed as follows:

$\text{SSIM}=(\frac{2\mu_{\text{x}}\mu_{\text{y}}+c_{1}}{\mu_{\text{x}}^{2}+\mu_{\text{y}}^{2}+c_{1}})(\frac{2\sigma_{\text{xy}}+c_{2}}{\sigma_{\text{x}}^{2}+\sigma_{\text{y}}^{2}+c_{2}})$ (S15)

where *μ*_x_ and *μ*_y_ represent the mean values of the generated and the target image, respectively. *σ*_x_ and *σ*_y_ represent the standard deviation of the generated and the target image, respectively. *σ*_xy_ represents the covariance of the generated and the target image. *c*_1_ and *c*_2_ are two constant terms that are used to prevent the denominator from being zero.

**S5.3: Dynamic holographic 3D AR reconstruction**

In order to verify that the proposed holographic camera can be utilized for the holographic 3D AR reconstruction, the related experiments are carried out as shown in Fig. S11. A real 3D scene consisting of the “butterfly”, “bird”, “black rabbit” and “black ladybug” signs are used in the experiment. The actual spacing between the signs is 50 mm. To shorten the optical path length, the spacing of the signs is set to 10 mm in the calculation of the hologram of the real 3D scene. Meanwhile, the recording distances of the signs are set to 300 mm, 310 mm, 320 mm, and 330 mm, respectively. The resolutions of both the real 3D scene and the depth map are 990×1760. In the experiment, only the hologram of the green channel of the real 3D scene is calculated and the calculation time is 53 ms. The markers “B”, “H”, “brown rabbit”, and “color ladybug” are used as references, which are located at the same depth plane as the “butterfly”, “bird”, “black rabbit”, and “black ladybug” signs, respectively.

As shown in Fig. S11a, the reconstructed image of the real 3D scene is shown in the red box, and the marker “H” and “butterfly” sign which are in the same depth plane are shown in the blue box. Currently, the camera is focused at a position 400 mm behind lens II. The enlarged reconstructed image of the “butterfly” sign is also shown in Fig. S11a. As shown in Fig. S11b, the reconstructed image of the real 3D scene is shown in the red box, and the marker “B” and “bird” sign which are in the same depth plane are shown in the blue box. Currently, the camera is focused at a position 410 mm behind lens II. The enlarged reconstructed image of the “bird” sign is also shown in Fig. S11b. As shown in Fig. S11c, the reconstructed image of the real 3D scene is shown in the red box, and the marker “brown rabbit” and “black rabbit” sign which are in the same depth plane are shown in the blue box. Currently, the camera is focused at a position 420 mm behind lens II. The enlarged reconstructed image of the “black rabbit” sign is also shown in Fig. S11c. As shown in Fig. S11d, the reconstructed image of the real 3D scene is shown in the red box, and the marker “color ladybug” and “black ladybug” signs which are in the same depth plane are shown in the blue box. Currently, the camera is focused at a position 430 mm behind lens II. The enlarged reconstructed image of the “black ladybug” sign is also shown in Fig. S11d.


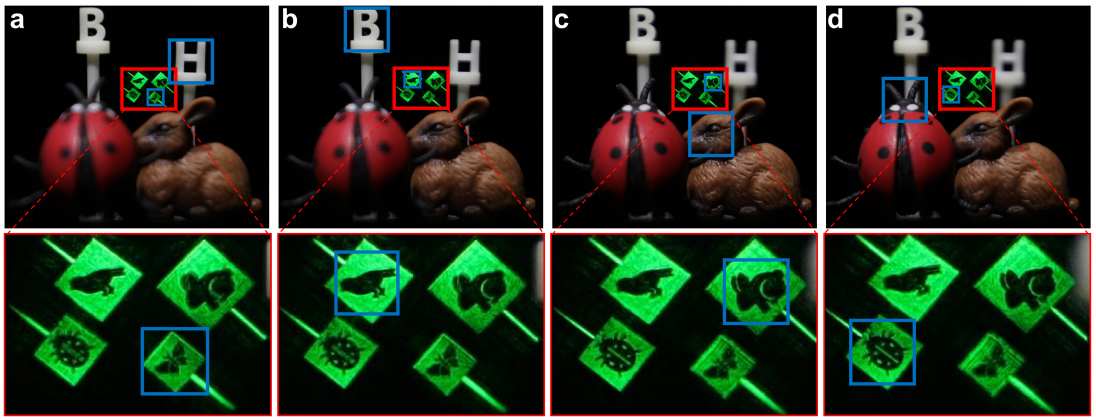


**Fig. S11 Experimental results of the holographic 3D AR reconstruction. a** Reconstructed image when focusing on the marker “H” and the “butterfly” sign. **b** Reconstructed image when focusing on the marker “B” and the “bird” sign. **c** Reconstructed image when focusing on the marker “brown rabbit” and the “black rabbit” sign. **d** Reconstructed image when focusing on the marker “color ladybug” and the “black ladybug” sign.

The dynamic reconstruction results are shown in Fig. S12. A movable “sun” sign is used to construct a real dynamic 3D scene. The liquid camera is used to capture the entire process of moving the “sun” sign from far to near. The total movement distance of the “sun” sign is 86 mm. The liquid camera is controlled to take a picture of the “sun” sign every 2 mm of its movement. In the end, the real dynamic 3D scene consists of 44 frames of the “sun” sign at different locations in space. When calculating the holograms of the real 3D dynamic scene, every 11 frames of the “sun” sign are considered to be in the same plane to divide the real dynamic 3D scene into 4 planes. Meanwhile, the benchmark recording distance is set to 300 mm and the interval between each plane is set to 10 mm. Therefore, the recording distances of the four planes of the real dynamic 3D scene composed of the “sun” sign are 300 mm, 310 mm, 320 mm, and 330 mm, respectively. As shown in Figs. S12a–d, the markers “tower”, “red bird”, “brown rabbit” and “bridge” are placed at a spatial interval of 10 mm. As the “sun” sign gradually moves from the marker “tower” to the marker “bridge”, the size of the “sun” sign changes from small to large. It can be seen from the results that high-quality dynamic holographic 3D reconstruction can be realized.

**
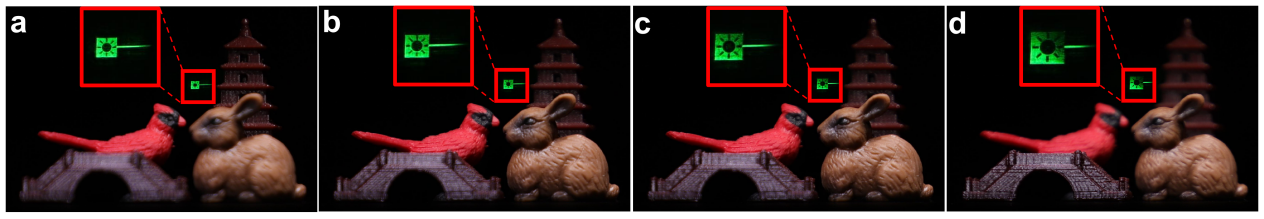
**

**Fig. S12 Experimental results of the dynamic holographic 3D reconstruction. a–d** Reconstructed images of the “sun” sign are in the same depth plane as the markers “tower”, “red bird”, “brown rabbit” and “bridge” respectively.

**S5.4: Training process and loss curves of the EEPMD-Net**

The training loss curve and validation loss curve of the model are shown in Fig. S13. As shown in Fig. S13a, at the end of the first stage, the training loss value of the red channel is 0.6157 and the validation loss value is 0.5447. The training loss value of the green channel is 0.5755 and the validation loss value is 0.5037. The training loss value of the blue channel is 0.5119 and the validation loss value is 0.4244. As shown in Fig. S13b, at the end of the second stage, the training loss value of the red channel is 0.7905 and the validation loss value is 0.8226. The training loss value of the green channel is 0.6811 and the validation loss value is 0.7204. The training loss value of the blue channel is 0.5852 and the validation loss value is 0.6045.


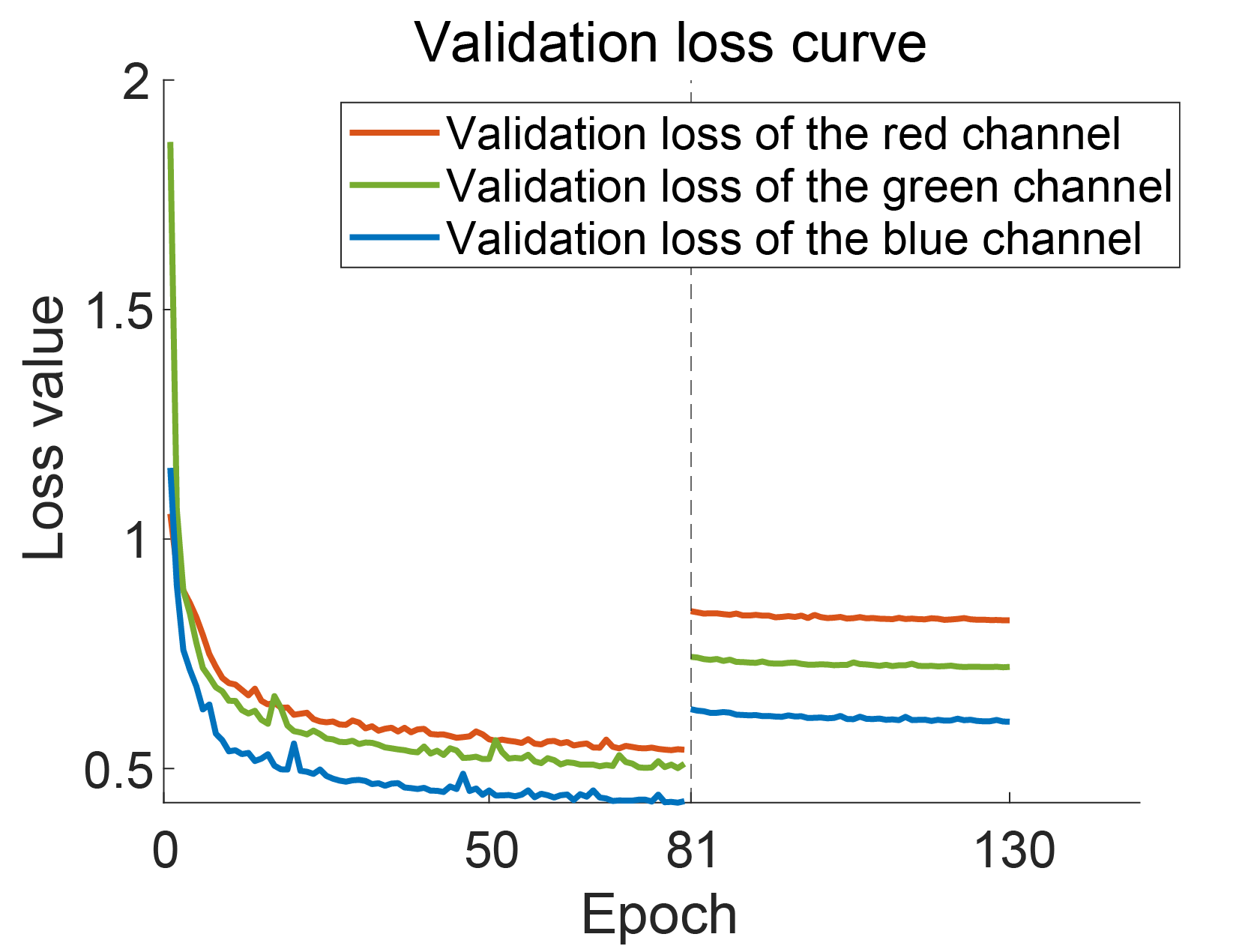

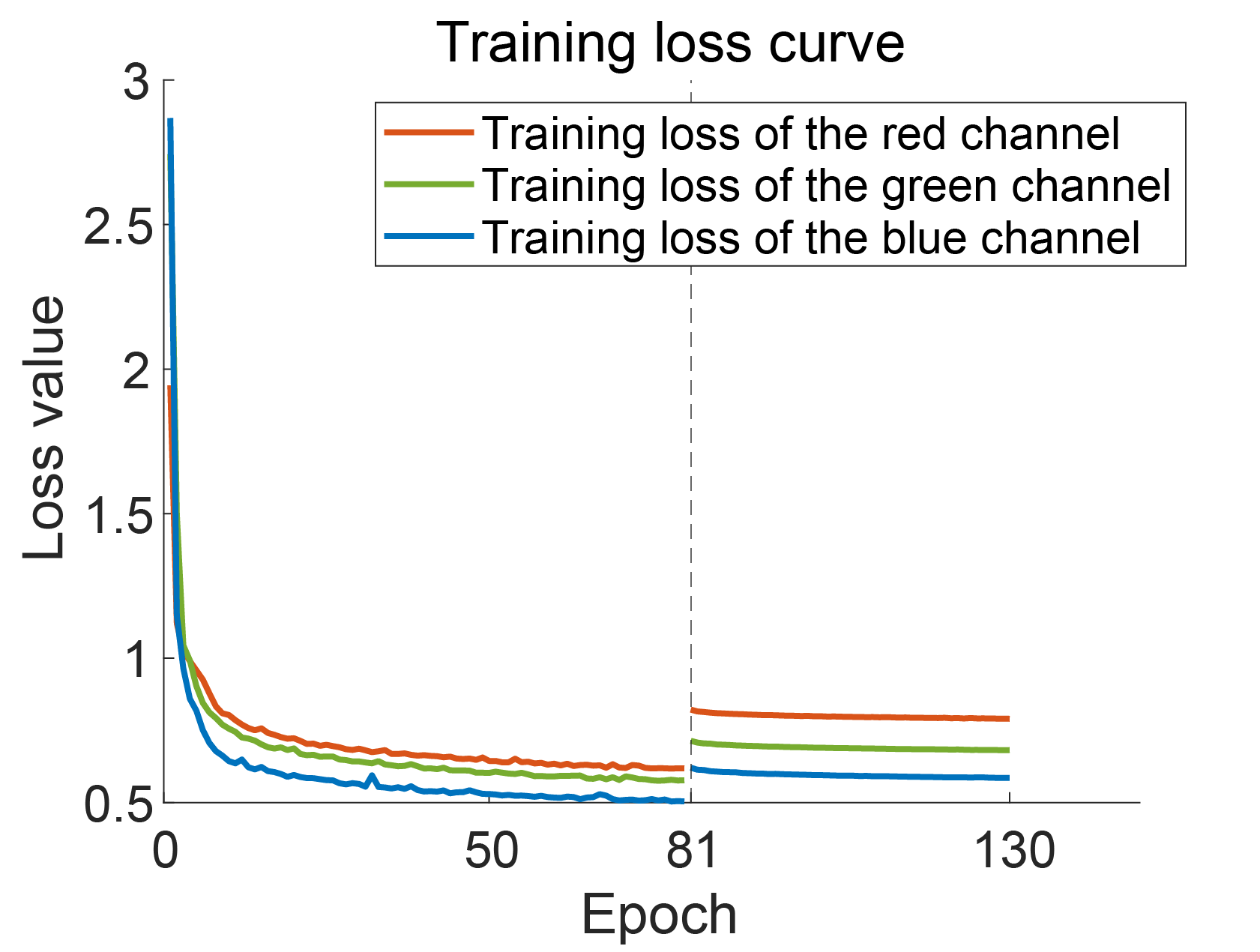


**a**

**b**

**Fig. S13 Loss curves of the EEPMD-Net. a** Training loss curve. **b** validation loss curve.

**References**

1. Zhuo, S. & Sim, T. Defocus map estimation from a single image. *Pattern Recogn.* **44**, 1852–1858 (2011).
2. Levin, A., Lischinski, D. & Weiss, Y. A Closed Form Solution to Natural Image Matting. In *Proc. IEEE Conference on Computer Vision and Pattern Recognition*, 61–68 (IEEE, 2006).
3. Li, Z. S., Zheng, Y. W., Li, Y. L., Wang, D. & Wang, Q. H. Method of color holographic display with speckle noise suppression. *Opt. Express* **30**, 25647–25660 (2022).
